# The words of our inner worlds: emotional language use in mind-wandering and dream reports reflects mental ill-being

**DOI:** 10.1093/nc/niag075

**Published:** 2026-10-31

**Authors:** Nanna Strid, Jarno Tuominen, Ryan Bernstein, Manuela Kirberg, Katja Valli, Jennifer Windt, Tristan Bekinschtein, Valdas Noreika, Antti Revonsuo, Pilleriin Sikka

**Affiliations:** Department of Psychology and Speech-Language Pathology and Turku Brain and Mind Center, University of Turku, Assistentinkatu 7, 20500 Turku, Finland; Department of Cognitive Neuroscience and Philosophy, University of Skövde, Högskolevägen 3, 54955 Skövde, Sweden; Department of Psychology and Speech-Language Pathology and Turku Brain and Mind Center, University of Turku, Assistentinkatu 7, 20500 Turku, Finland; Department of Psychology, Stanford University, 450 Jane Stanford Way, Building 420, Stanford, 94305 CA, United States; Department of Philosophy, Monash University, 20 Chancellor's Walk, Clayton, 3800 VIC, Australia; Monash Centre for Consciousness and Contemplative Studies, Monash University, 4th Floor/29 Ancora Imparo Way, Clayton, 3800 VIC, Australia; Department of Psychology and Speech-Language Pathology and Turku Brain and Mind Center, University of Turku, Assistentinkatu 7, 20500 Turku, Finland; Department of Cognitive Neuroscience and Philosophy, University of Skövde, Högskolevägen 3, 54955 Skövde, Sweden; Department of Philosophy, Monash University, 20 Chancellor's Walk, Clayton, 3800 VIC, Australia; Monash Centre for Consciousness and Contemplative Studies, Monash University, 4th Floor/29 Ancora Imparo Way, Clayton, 3800 VIC, Australia; Consciousness and Cognition Lab, Department of Psychology, University of Cambridge, CB2 3EB Cambridge, United Kingdom; Department of Biological and Experimental Psychology, School of Biological and Behavioural Sciences, Queen Mary University of London, G.E. Fogg Building, Mile End Road, E1 4NS London, United Kingdom; Department of Psychology and Speech-Language Pathology and Turku Brain and Mind Center, University of Turku, Assistentinkatu 7, 20500 Turku, Finland; Department of Cognitive Neuroscience and Philosophy, University of Skövde, Högskolevägen 3, 54955 Skövde, Sweden; Department of Psychology and Speech-Language Pathology and Turku Brain and Mind Center, University of Turku, Assistentinkatu 7, 20500 Turku, Finland; Department of Cognitive Neuroscience and Philosophy, University of Skövde, Högskolevägen 3, 54955 Skövde, Sweden; Department of Psychology, Stanford University, 450 Jane Stanford Way, Building 420, Stanford, 94305 CA, United States; Department of Anesthesiology, Perioperative and Pain Medicine, School of Medicine, Stanford University, 300 Pasteur Drive, Stanford, 94305 CA, United States

**Keywords:** spontaneous thought, dreaming, mind-wandering, LIWC, emotions, language

## Abstract

Do the words we use reveal how we feel? While much research has explored language use in social media, little is known about how the content of spontaneous thoughts and experiences, such as daytime mind-wandering and nighttime dreaming, reflects well-being, and ill-being. We analyzed 1 496 mind-wandering reports (*N* = 152) and 1781 dream reports (*N* = 172) using the Linguistic Inquiry and Word Count. Participants also self-reported their well-being and ill-being. Linear mixed-effects models showed that ill-being (negative affect, anxiety, and depression symptoms) was associated with the use of negative tone and negative emotion words (including anxiety, anger, sadness words) in mind-wandering reports. Ill-being (specifically, negative affect) was also associated with greater use of anxiety words in dream reports. Well-being (eudaimonic well-being and peace of mind) was associated with the use of positive tone words in mind-wandering reports, but this association was not robust in sensitivity analyses. These findings indicate that the way people describe their feelings in reports of different conscious states reflects aspects of their mental health and may contribute to future diagnostic tools in psychology and psychiatry.

## Introduction

Mental ill-being, including anxiety and depression, affects nearly 600 million people globally, yet mental health services remain critically underfunded worldwide (World Health Organization 2022, Gallup 2023). This underscores the urgent need for reliable, scalable, and cost-effective diagnostic and prognostic markers for mental health. Recent research suggests that the language people use can effectively track and predict mental health outcomes (Nook et al. 2022, Nook 2023). For example, negative emotion words expressed in social media posts, personal communication*,* and clinical settings—assessed through tools like the Linguistic Inquiry and Word Count (LIWC; Pennebaker et al. 2022)—have been linked to symptoms of affective disorders (Settanni and Marengo 2015; Guntuku et al. 2017; Eichstaedt et al. 2018, 2021; Sonnenschein et al. 2018; Corbin et al. 2023; Stade et al. 2023). Furthermore, linguistic markers have been shown to track treatment progress in real-world therapeutic interactions (Nook et al. 2022). However, it remains unclear whether emotional language in reports of spontaneous thoughts and experiences can serve as reliable markers of mental health.

Spontaneous thoughts and experiences, such as mind-wandering (or daydreaming) during wakefulness and dreaming during sleep, are mental states or sequences of mental experiences where thoughts or images flow freely, largely unconstrained by external stimuli or cognitive control (Christoff et al. 2016). Adults spend a third to half of their time engaged in mind-wandering (Klinger and Cox 1987; Killingsworth and Gilbert 2010; Kane et al. 2017), and dreaming is a near-universal phenomenon during sleep (Pagel 2003). As such, these experiences provide a largely unfiltered view of people’s inner lives and emotional states. While both mind-wandering and dreaming offer a unique window into mental well-being, the relationship between mind-wandering and dreaming is still debated. Some theories suggest a continuity between them, proposing that dreaming is an intensified form of mind-wandering (Schredl 2003; Fox et al. 2013; Klinger 2013; Domhoff 2018; Windt 2021). According to the continuity perspective, mind-wandering, and dreaming share underlying mechanisms and reflect ongoing thoughts, concerns, and emotions that persist across waking and sleep states. For example, according to the dynamic framework of thought, conscious experiences exist on a continuum (from active wakefulness, to mind-wandering, to night-time dreaming) and differ from each other due to their various levels of constraint. The content and dynamics of mental states are constrained either deliberately *via* cognitive control or automatically *via* affective and sensory salience. In this view, mind-wandering is a form of spontaneous thought whose contents and transitions are more deliberately constrained than those of dreaming, but less so than those of ruminative, task-centric, creative, and obsessive thought. Dreams, then, are characterized by affect that is unconstrained by external stimuli (Christoff et al. 2016; Windt 2021). Conversely, the discontinuity perspective proposes that dreaming involves different cognitive and emotional processes compared to mind-wandering (Hobson et al. 2000; Voss et al. 2011).

Although both mind-wandering and dreaming are marked by varying degrees of emotion and have been linked to mental well-being and ill-being (Sikka et al. 2017, 2018a, 2021; Kam et al. 2024), few studies have directly examined how emotional content in both states reflects mental health. Studying both mind-wandering and dreaming allows us to explore whether the emotional content of these experiences is similarly or differently associated with well-being and ill-being, providing insights into the continuity or discontinuity of our experiences across different states.

Mental health refers to not only the absence of mental ill-being but also the presence of mental well-being (Keyes 2005; Ryff et al. 2006). The umbrella term well-being encompasses both hedonic well-being (feeling good and being satisfied with one’s life), eudaimonic well-being (personal growth and a sense of purpose; Ryan and Deci 2001; Ryff et al. 2006), and the more recently integrated concept peace of mind (inner peace and harmony; Lee et al. 2013; Sikka et al. 2018b, 2023a). In contrast, mental ill-being involves states of distress and dysfunction, typically manifesting as negative affect, anxiety, or depression (Diener 2006; Ryff et al. 2006). Understanding how the emotional content of spontaneous thoughts and experiences reflects these aspects of well-being and ill-being may offer valuable pathways for understanding and assessing changes in mental health.

Research suggests that the content of spontaneous thoughts and experiences can reveal patterns of emotional language related to mental health. For example, Vine et al. (2020) developed and used a text analysis software based on LIWC to analyze active emotion vocabularies expressed in stream-of-consciousness essays and blogs. They found that people with greater differentiation in negative emotion vocabularies showed higher levels of ill-being, whereas those with more differentiated positive emotion vocabularies exhibited lower levels of ill-being. Similarly, using LIWC to code naturally occurring, unprompted thoughts, Raffaelli et al. (2021) reported that rumination, a key feature of ill-being, was associated with more negative, past-oriented, and self-focused thoughts. Parallel findings in dream research show that negative affect expressed in dream reports correlates with anxiety, depression, and overall psychopathology (Pesant and Zadra 2006; King and DeCicco 2007; Sikka et al. 2018b; Rimsh and Pietrowsky 2021), while positive affect in dream reports has been linked to peace of mind (Sikka et al. 2018b). Using graph analysis, Mota et al. (2014) analyzed dream reports and transcribed interviews of schizophrenic, bipolar, and healthy individuals, finding that dream reports mirrored patients’ mental states more accurately than interviews. More recently, Mota et al. (2020) applied LIWC to dream reports and found that the emotional language in these reports reflected participants’ mental distress during the COVID-19 pandemic.

Given how common mind-wandering and dreaming are in daily life, exploring their potential for monitoring mental health is crucial. If reports of spontaneous thoughts and experiences can reliably reflect well-being and ill-being, they could become valuable tools for assessing mental health. This could be especially impactful in clinical settings, offering scalable, and cost-effective tools to discreetly track mental health states and intervene in affect-related psychopathology. In this context, the growing field of digital psychiatry, coupled with linguistic analysis tools like LIWC, could harness these spontaneous reports as part of large-scale mental health solutions.

In this study, we investigated whether the emotional language in written mind-wandering and dream reports reflects well-being and ill-being. We hypothesized that (1) well-being measures (life satisfaction, domain satisfaction, positive affect, eudaimonic well-being, and peace of mind) would be positively associated with the use of positive emotion words in mind-wandering and dream reports, but negatively associated with the use of negative emotion words in these reports; (2) ill-being measures (negative affect, anxiety, and depression) would be negatively associated with the use of positive emotion words in mind-wandering and dream reports, but positively associated with the use of negative emotion words. Consistent with prior findings (Sikka et al. 2018b), we expected peace of mind and anxiety to be the strongest predictors of positive and negative vocabularies, respectively.

To test these hypotheses, we analyzed the daily diary data collected in the UK and Australia over a 4-month period (July—November 2020; Sikka et al. 2023b). Briefly, participants first completed a questionnaire measuring various aspects of well-being and ill-being. After this, over the course of 2 weeks, participants completed two daily diaries. In the evenings, they engaged in a 10-min mind-wandering session and subsequently filled out a mind-wandering diary, reporting everything that went through their minds during the session. In the mornings, immediately upon awakening, participants filled out dream diaries, reporting their dreams from the previous night (see Fig. 1). We applied LIWC to the 1496 mind-wandering reports from 152 individuals and 1781 dream reports from 172 individuals to examine how well-being and ill-being measures predicted the emotional language used in those reports (measured as relative word frequencies within emotion word categories). Based on principal component analysis, the main analyses were conducted using well-being and ill-being composites (calculated as the mean of standardized scores of its constituent measures).

**Figure 1:**
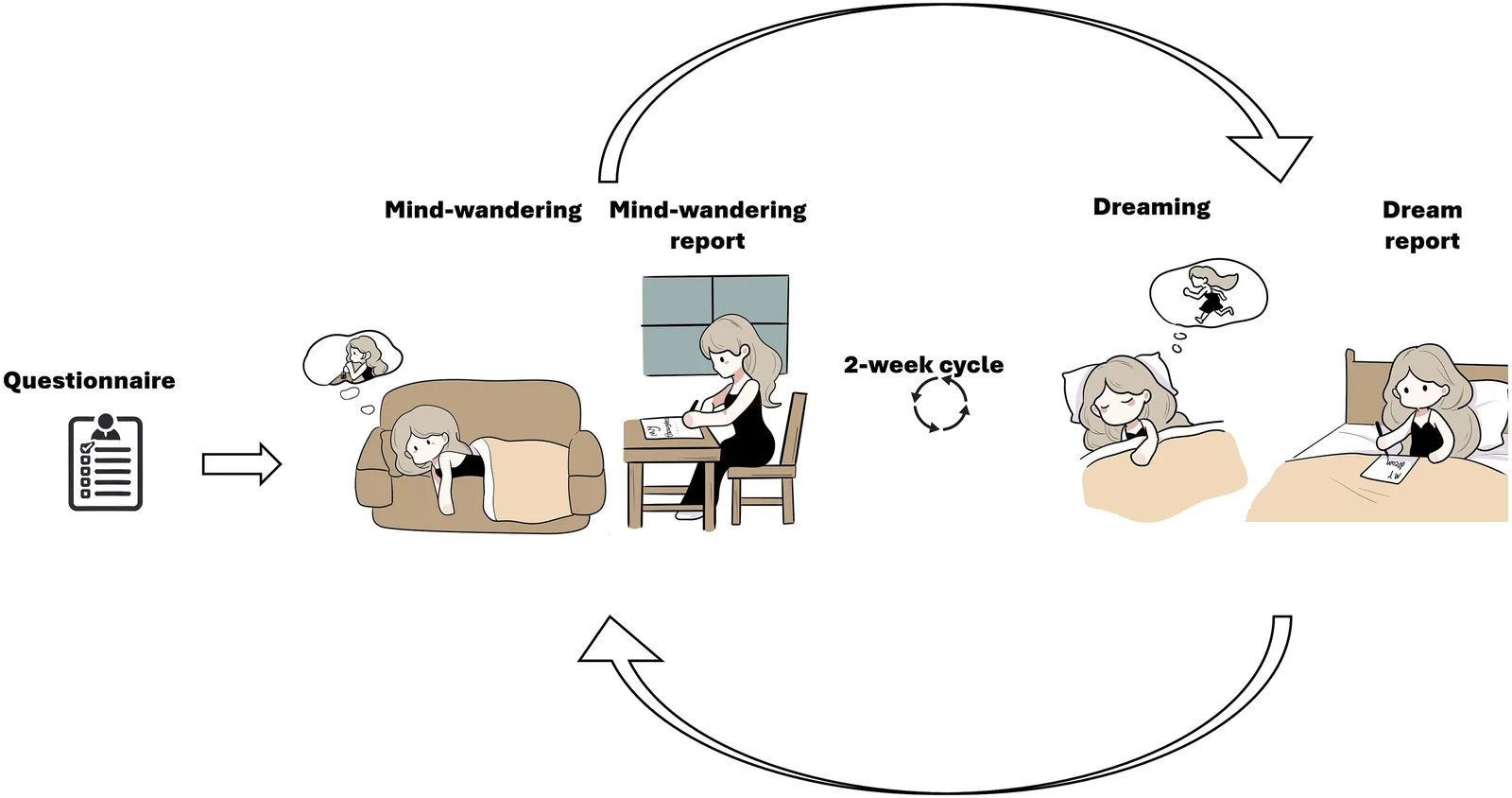
An overview of the data collection procedure*.* Participants completed a questionnaire measuring aspects of well-being (life satisfaction, domain satisfaction, positive affect, eudaimonic well-being, and peace of mind) and ill-being (negative affect, symptoms of depression, and anxiety). Then, every day for the next 2 weeks, participants engaged in a 10-min mind-wandering session, after which they provided a free-form written report of the content of their experiences during the session. Additionally, each morning during the 2 weeks, participants provided free-form written reports of any dreams they had the previous night.

## Methods and materials

### Participants and procedure

This study analyzed data collected in the framework of a large international study, ‘The MIND Research Study’ (https://osf.io/su7mn/). Participants were recruited *via* press releases and advertisements in social media. A total of 1781 dream reports were collected from 172 participants (139 women, 25 men, 8 ‘other’; ages 19–78, *M* = 46.99, *SD* = 14.80 years), 64 Australian and 108 British. Additionally, 1496 mind-wandering reports were collected from 152 participants (123 women, 23 men, 6 ‘other’; ages 19–78, *M* = 48.42, *SD* = 14.71 years), 59 Australian and 93 British. Of the 172 participants who provided dream reports, 148 also provided mind-wandering reports, while four participants provided mind-wandering reports but no dream reports.

All data were collected online during 4 months (July to November 2020) *via* a website created for the purposes of this study. Participants were prompted to read through the study description and instructions and then received a unique participant ID to use throughout the study. After providing informed consent and confirmation of being at least 18 years old, participants completed a questionnaire containing demographic questions and a battery of scientifically validated well-being and ill-being scales (see Materials and Measures). Following this, they provided mind-wandering and dream reports daily for 14 days (see Fig. 1). Participants received no reimbursement for participation. Ethical approval was obtained from the Cambridge Psychology Research Ethics Committee and the Monash University Human Research Ethics Committee. The authors assert that all procedures contributing to this work comply with the ethical standards of the relevant national and institutional committees on human experimentation and with the Helsinki Declaration of 1975, as revised in 2008.

## Materials and measures

### Well-being and ill-being scales

Mental well-being was conceptualized as consisting of hedonic well-being (life satisfaction, domain satisfaction, and positive affect), eudaimonic well-being, and peace of mind; ill-being as the presence of negative affect, and symptoms of anxiety and depression. Empirically validated well-being and ill-being scales were administered online in English.

#### Life satisfaction

The Satisfaction with Life Scale (SWLS; Diener et al. 1985) was used to measure participants’ global life satisfaction. This well-validated and highly reliable scale is an established measure of life satisfaction (Pavot and Diener 1993). The scale prompts participants to rate five statements (e.g*.* ‘I am satisfied with my life’) from 1 (strongly disagree) to 7 (strongly agree). The summed five items provided the total score, with higher scores denoting higher life satisfaction. See Table 1 for descriptive statistics and internal consistency (Cronbach’s α) for this and all other well-being and ill-being measures.

**Table 1:** Reliability and descriptive statistics of well- and ill-being measures (N = 176)

WB or IB	Aspect of WB & IB	Scale	Cronbach’s alpha	*M* (*SD*)	*Mdn*	Observed range (Min-Max)	Theoretical range (Min-Max)
WB	Life Satisfaction	SWLS	0.89	22.66 (7.29)	24.00	5–35	5–35
	Domain Satisfaction	BBQ	0.74	62.35 (18.15)	63.00	3–96	0–96
	Positive Affect	PANAS-PA	0.89	32.02 (8.22)	32.00	12–50	10–50
	Eudaimonic WB	PWB	0.81	95.64 (13.31)	96.00	55–122	18–126
	Peace of Mind	PoM-5	0.89	2.40 (0.56)	2.40	1–3.8	1–4
	WB Composite	Well-Being	0.85	43.01 (7.76)	43.48	16.24–59.36	6.8–62.2
IB	Depression	PHQ-9	0.84	6.74 (4.91)	6.0	0–26	0–27
	Anxiety	GAD-7	0.90	5.63 (4.91)	4.5	0–21	0–21
	Negative Affect	PANAS-NA	0.87	22.30 (7.74)	22.00	10–43	10–50
	IB Composite	Ill-Being	0.86	11.56 (5.21)	10.42	3.33–27.50	3.33–32.67

*Note.* Includes 172 participants with dream logs (of whom 148 provided mind-wandering logs) plus 4 participants who provided mind-wandering logs but not dream logs (152 participants with mind-wandering logs in total). Cronbach’s *α* was based on 172 participants for individual scales and 176 for the composite scores. IB = ill-being, WB = well-being. LIWC = Linguistic Inquiry and Word Count. *N* = number of participants. SWLS = Satisfaction with Life Scale. BBQ = Brunnsviken Brief Quality of Life Scale (BBQ). PANAS = Positive Affect Negative Affect Schedule. PA = positive affect. NA = negative affect. PWB = Ryff’s Scales of Psychological Well-Being. PoM-5 = Peace of Mind Scale. PHQ-9 = Depression scale from Patient Health Questionnaire. GAD-7 = Generalized Anxiety Disorder Scale.

#### Domain satisfaction

The Brunnsviken Brief Quality of Life Scale (BBQ; Lindner et al. 2016) assessed participants’ satisfaction with major life domains. On this 12-item scale, participants were asked to rate how satisfactory (e.g*.* ‘I am satisfied with my learning: I have the opportunity and desire to learn new, exciting things and skills that interest me.’) and important (e.g*.* ‘Learning is important for my quality of life’) they currently considered six life domains (leisure time, view on life, creativity, learning, friends and friendship, view of self) to be, from 0 (do not agree at all) to 4 (agree completely). The total score was calculated by multiplying the satisfaction and importance rating for each domain, then summing their products.

#### Positive and negative affect

Positive affect was measured *via* the ten positive emotion items (e.g*.* enthusiastic, proud, active) on the Positive and Negative Affect Schedule (PANAS-PA; Watson et al. 1988), which is among the most used and highly validated measures of affect (Crawford and Henry 2004). Negative affect was measured *via* the ten negative emotion items (e.g*.* scared, irritable, jittery) of PANAS (PANAS-NA). Participants rated to what extent (1: very slightly or not at all, to 5: extremely) they had experienced each of the twenty emotions within the past 2 weeks. The summed positive and negative emotion items yielded the scores on the positive and negative affect subscales, respectively.

#### Eudaimonic well-being

Eudaimonic well-being was measured *via* the 18-item Psychological Well-Being Scale (PWB; Ryff and Keyes 1995). The scale is subdivided into six subscales: autonomy (e.g*.*, ‘I judge myself by what I think is important, not by the values of what others think is important.’), environmental mastery (e.g*.* ‘In general, I feel I am in charge of the situation in which I live.’), personal growth (e.g*.* ‘For me, life has been a continuous process of learning, changing, and growth.’), positive relations with others (e.g*.* ‘People would describe me as a giving person, willing to share my time with others.’), purpose in life (e.g*.* ‘Some people wander aimlessly through life, but I am not one of them.’), and self-acceptance (e.g*.* ‘I like most parts of my personality.’), with three items for each subscale. Participants rated their agreement with each statement, from 1 (strongly agree) to 7 (strongly disagree). The summed items provided the total score, with higher scores signifying greater eudaimonic well-being.

#### Peace of mind

Inner peace and harmony were measured *via* the Peace of Mind Scale (PoM; Lee et al. 2013). The scale consists of seven items, two of which are reverse-scored. Participants indicated how often they felt inner peace and ease in their daily lives by rating each statement (e.g*.* ‘My mind is free and at ease’) from 1 (not at all) to 4 (all of the time) (unlike the original scale, the scale we used did not include the middle scale point alternative ‘often’). Previous studies have shown that, in Western samples, the two reverse-scored items load on a separate factor (Sikka et al. 2018b, 2023a). We performed a confirmatory factor analysis using the *lavaan* function (Rosseel 2012) in R. As previously reported, the 2-factor PoM solution was a significantly better fit (*CFI* = .93, *RMSEA* = .139, *SRMR* = .057) than the 1-factor solution (*CFI* = .90, *RMSEA* = .161, *SRMR* = .067), *χ2* = 19.986, *P* < .001. As such, we used the 5-item PoM (i.e*.* PoM-5), containing all non-reversed items, in our analyses.

#### Depression

Depressive symptoms were assessed using the 9-item Patient Health Questionnaire (PHQ-9; Kroenke et al. 2001). The scale draws from the diagnostic criteria in the Diagnostic and Statistical Manual of Mental Disorders (DSM-5; American Psychiatric Association 2013). Participants indicated how frequently, over the past 2 weeks, they had been bothered by each of nine symptoms (e.g*.* ‘Feeling down, depressed, or hopeless’), from 0 (not at all) to 3 (nearly every day). The summed scores reflect the level of depression, with higher scores denoting more severe depression (scores 5, 10, 15, and 20 denote mild, moderate, moderately severe, and severe depression, respectively).

#### Anxiety

Symptoms of anxiety were measured *via* the seven items on the Generalized Anxiety Disorder Scale (GAD-7; Spitzer et al. 2006). The measure is based on the first three (A, B, C) diagnostic criteria for generalized anxiety disorder in the DSM-5. Participants indicated how often they had been bothered by seven problems (e.g*.* ‘Feeling nervous, anxious or on edge’) over the past 2-week period, from 0 (not at all) to 3 (nearly every day). The summed scores reflect the level of anxiety, with higher scores implying more severe anxiety (scores 5, 10, and 15 represent mild, moderate, and severe anxiety, respectively).

### Mind-wandering reports

Mind-wandering was first defined in the instructions to participants as ‘the contents that enter our mind when we are not engaged in or focused on any specific activity or task, or when such activities/tasks are sufficiently automatized. For example, our minds may wander when we go to bed in the evenings, while we brush our teeth, or while driving somewhere.’ For the mind-wandering task, participants were instructed to let their minds freely wander for a 10-min period each evening, ideally around the same time. They were encouraged to refrain from trying to control their thoughts, and to set a timer to avoid having to monitor the time. They were asked to perform this task in a quiet place free of disturbances, with their eyes open or closed. Promptly following the 10 min, participants were asked to write down, in as much detail as possible and without censoring any content, everything that had gone through their minds during the 10-min mind-wandering period. Finally, they rated on a 5-point scale (1: not at all, to 5: extremely) the extent to which they had experienced positive and negative affect during mind-wandering.

### Dream reports

Participants were instructed to complete a dream log immediately upon awakening each morning, and to do so every day, including on mornings when no dream was recalled. They first indicated whether they remembered any dream experiences from the preceding night, choosing between recalling a dream including at least some of its content, believing they had dreamt but recalling nothing of it (a so-called ‘white dream’), or believing they had not dreamt. Those who reported recalling dream content were asked to report the dreams ‘exactly as [they had] experienced them,’ and to ‘report everything [they] remember (what happened, where, who was present, what [they] were thinking and feeling): no detail is too irrelevant or inconsequential to be reported.’ Participants were also asked not to censor the report or make the dream more structured, organized, logical, or complete than how they remembered it. Participants who woke during the night and recalled a dream were instructed to record it at that point rather than waiting until morning.

Upon enrolling in the study, participants received detailed instructions on how to remember and report their dreams, including keeping the log within reach, forming a pre-sleep intention to recall their dreams, remaining still upon awakening and mentally replaying the dream, briefly noting the main events in bullet points, and then writing down the report in full. No formal definition of ‘dream’ was provided. Participants relied on their everyday understanding of the term, guided by the content specification above. This was a deliberate choice, intended to avoid constraining which experiences participants judged reportable and thereby excluding fragmentary or thought-like experiences of theoretical relevance to the comparison with mind-wandering. Participants were asked to submit one dream per report and to re-open the reporting form for each additional dream from the same night. They were also asked to provide any waking commentary (such as contextualization of reported events) in brackets. Participants also rated on a 5-point scale (1: not at all, to 5: extremely) the extent to which they had experienced positive and negative affect during the dream. Although this study primarily focused on the analysis of narrative mind-wandering and dream reports, supplementary analyses were performed to examine the convergence between participants’ self-ratings of affect and corresponding LIWC emotional language categories.

The full reporting instructions for the mind-wandering and dream logs can be found on https://osf.io/su7mn/overview. Although participants were given clear instructions regarding when to perform the mind-wandering and dream tasks, there were no practical constraints set by the researchers on when they could complete the tasks and submit the reports within the data collection period. All mind-wandering and dream reports were collected in written form only.

## Data analysis

### Emotional language use in mind-wandering and dream reports

First, all the mind-wandering and dream reports were pruned by two coders (NS and RB). The process involved marking and removal of all waking commentary not part of the actual experience from the reports. Waking commentary provided in brackets (as per instructions), as well as text that was clearly waking commentary but not provided in brackets, was removed. For example, in the following excerpts the italicized parts were removed: ‘*(this is a small part of a longer dream, I suspect)* I am in a classroom together with my husband…’ and ‘*this was my second dream of the night.* I was in a huge house which I owned…’ In cases where text in brackets was clearly part of the dream, that text was retained in the report. Reports that contained multiple separate dreams were separated so that each report contained only one dream.

Lemmatization was not implemented because LIWC already accounts for different forms of many words through the use of word stems marked with asterisks (Boyd et al. 2022). Stop-word removal was also not applied because, in standard LIWC dictionary analyses, words are retained and matched against the predefined dictionaries. Removing stop words could therefore remove words included in LIWC categories being analyzed. Stop-word removal is primarily relevant to other linguistic methods, such as meaning-extraction and open-vocabulary approaches. We therefore retained the original text, apart from pruning described above, to preserve comparability with LIWC’s standard dictionary-based scoring procedure.

Emotional language use was analyzed using the Linguistic Inquiry Word Count (LIWC-22, v. 1.2.0) text analysis software (Pennebaker et al. 2022). The default internal LIWC-22 Dictionary (English) was applied. All words in the narrative reports directly associated with affect or emotion within LIWC-22 (i.e*.* all available affect-related categories) were analyzed. This involved the LIWC-22 ‘affect’ category (2999 words), with its subordinate categories ‘positive tone’ (e.g*.* well, new, love), ‘negative tone’ (e.g*.* bad, wrong, too much), and ‘emotion.’ Within ‘emotion,’ ‘positive emotion’ (e.g*.* good, happy, hope) and ‘negative emotion’ (e.g*.* bad, hurt, tired), and subcategories of the latter, ‘anxiety’ (e.g*.* worry, fear, nervous), ‘anger’ (e.g*.* hate, mad, frustr^*^), and ‘sadness’ (e.g*.* disappoint^*^, cry), were selected. Each category’s original vocabulary was used as-is, and hence no words or phrases were added to or removed from any of the categories. Because the umbrella categories ‘affect’ (first level) and ‘emotion’ (second level) did not contribute insights beyond those already provided by their subcategories, these were left out of the final analysis. Thus, seven subcategories were analyzed. In addition, word count was summarized using LIWC-22.

In LIWC-22, positive and negative tone categories reflect sentiment and contain words broadly associated with positive and negative emotion (happy, joy, sad, etc.), words associated with those emotions (beautiful, birthday, funeral, etc.), and selected modifiers (e.g*.* too much). More narrowly, the categories ‘Positive emotion’ and ‘Negative emotion’ only include true emotion labels and words that strongly suggest emotion (e.g*.* ‘laughter,’ which typically implies positive affect). ‘Negative emotion’ includes numerous undifferentiated negative emotion words, of which subsets are contained within the narrower categories of ‘anxiety,’ ‘anger,’ and ‘sadness’ (Boyd et al. 2022). The score in each category represents the respective percentage of affect or emotion words out of the total number of words in the report.

### Statistical analyses

Statistical analyses were performed using R (v. 4.2.1; R Core Team 2020). Mind-wandering and dream reports were analyzed separately as not all participants overlapped. Because most of the variables were non-normally distributed, Spearman correlations were conducted to examine the relationship between well-being and ill-being scales and LIWC-22 categories.

To avoid issues with multicollinearity between the various well-being and ill-being variables, principal component analysis (PCA) was performed based on standardized scale scores (scores were mean-centered and scaled to unit variance; see Supplementary Fig. 1 and Supplementary Table 1). The first principal component showed a bipolar contrast between well-being and ill-being measures, while the second principal component reflected positive loadings across all eight scales. Based on the overall pattern of the loadings, the measures were grouped into theoretically defined well-being and ill-being composites. At the participant level, each individual scale score was standardized (z-scored), and the well-being and ill-being composite scores were calculated as the mean of the standardized scores of their respective constituent scales. The well-being composite consisted of SWLS, BBQ, PANAS-PA, PWB, and PoM-5, whereas the ill-being composite consisted of PANAS-NA, GAD-7, and PHQ-9. The resulting well-being and ill-being composite scores were moderately negatively correlated (r = −.47, *P* < .001). Participant-level composite scores were calculated and subsequently merged into both report-level datasets.

The emotional language outcome variables (i.e*.* LIWC-22 relative word frequencies within the specified categories) were continuous dependent variables that were positively skewed. We applied a square root transformation to the outcome variable to mitigate skewness. The transformed variable was then modeled using the lmer function from the lme4 package (Bates et al. 2015). Although normality of residuals was not perfectly achieved, we used these models as our primary analysis because they are robust to non-normal residuals at these sample sizes and because they provide parsimonious and interpretable models. To verify that the distributional assumption did not affect our conclusions, we refitted all primary models as Tweedie generalized linear mixed models (GLMM) on the untransformed outcomes using the function glmmTMB from the R package glmmTMB (Brooks et al. 2017). We did not directly compare the two model families by information criteria, because they are fitted to different response scales and their likelihoods are therefore not comparable.

LIWC-22 categories (positive tone, negative tone, positive emotion, negative emotion, anxiety, anger, sadness) were level-1 outcome variables, whereas well-being composite score and ill-being composite score were level-2 fixed-effect predictors that were standardized before model estimation. To control for age (continuous variable), gender (categorical variable: woman, man, other), and country (categorical variable: Australia, United Kingdom), these variables were added as level-2 fixed-effect covariates in all models. Age was standardized before model estimation. Gender and country were entered as categorical factors using treatment (dummy) coding, with woman serving as the reference category for gender and Australia for country. To account for the nested data, participant-specific random intercept was included in all models. Benjamini-Hochberg False Discovery Rate correction (FDR; Benjamini and Hochberg 1995) was then applied across the 28 focal tests of our hypotheses (2 predictors × 7 LIWC outcomes × 2 report types). Age, gender, and country were covariates and were not included in the FDR correction family because they were included to adjust the focal estimates rather than as hypotheses under test. As a sensitivity analysis, we repeated the primary models after excluding reports containing fewer than 25 words, given that LIWC-based analyses have recommended a minimum length of 25 words for more stable percentage-based estimates (Boyd 2017). The same 28-test FDR correction family was used in the sensitivity analyses.

In case a well-being or ill-being composite was a significant predictor of LIWC scores, we performed follow-up models in which we tested the particular well-being and ill-being variables that make up the respective composite as predictors. To avoid multicollinearity issues, each of these variables was entered separately in a model (together with the control variables age, gender, and country).

## Results

### Descriptive statistics

The original data contained 1767 mind-wandering reports and 2604 dream reports (Sikka et al. 2023b). Because the subsequent analyses used multilevel models, participants who provided at least three reports of the respective report type were included in the analyses (Arend and Schäfer 2019; Sikka et al. 2023b). Following this criterion, participants provided an average of *M* = 10.35 dream reports (*SD =* 6.10, *Mdn* = 9.00, range = 3–32) and *M* = 9.84 mind-wandering reports (*SD =* 5.28, *Mdn* = 10.00, range = 3–40) during their participation in the study. Because the study allowed participants to submit multiple dream reports from the same night, some participants provided a relatively high number (>30) of dream reports over the 14-day period. Additionally, although the protocol specified a 2-week logging period, participants who wished to continue were permitted to do so for longer. Tables 1 and 2 display the descriptive statistics and internal consistency (Cronbach’s *α*) of each well-being and ill-being scale and the associated composite variables, and of LIWC categories, respectively. The scores mirror the established normative values for each scale, indicating alignment with previous research.

**Table 2:** Descriptive statistics of LIWC categories

Type of reports	LIWC category	*M*	*SD*	*Mdn*	Observed range (Min—Max)
Mind-Wandering Reports (N = 152, n = 1496)	Word Count	193.94	141.86	164	3–1 160
	Positive Tone	3.07	2.18	2.79	0–25
	Negative Tone	2.28	2.29	1.83	0–20
	Positive Emotion	1.11	1.29	0.78	0–12.5
	Negative Emotion	1.38	1.74	0.95	0–16.67
	Anxiety	0.38	0.80	0.00	0–10.42
	Anger	0.24	0.62	0.00	0–10.53
	Sadness	0.20	0.60	0.00	0–10.87
Dream Reports (N = 172, n = 1781)	Word Count	181.11	164.13	137	1–1 346
	Positive Tone	1.59	1.73	1.27	0–25
	Negative Tone	1.49	1.85	1.04	0–21.74
	Positive Emotion	0.36	0.68	0.00	0–7.69
	Negative Emotion	0.73	1.20	0.31	0–13.04
	Anxiety	0.28	0.85	0.00	0–12.5
	Anger	0.15	0.50	0.00	0–11.11
	Sadness	0.11	0.48	0.00	0–12.5

*Note.* LIWC = Linguistic Inquiry and Word Count. *N* = number of participants, *n* = number of reports.

### Relationship between waking well-being and ill-being and emotional language use in mind-wandering and dream reports

Correlations between all the measures can be seen in Supplementary Tables 2–4. Linear mixed-effects models showed that, after controlling for multiple comparisons, ill-being predicted negative tone as well as the use of negative emotion words, including anxiety, anger, and sadness, in mind-wandering reports (see Table 3 and Fig. 2). Well-being predicted the positive tone of mind-wandering reports, although this was the weakest of the significant associations and was not robust in sensitivity analyses (see below). As for dream reports, measures of ill-being predicted the use of anxiety words (see Table 4 and Fig. 2).

**Table 3:** Results of linear mixed effects models predicting emotional language use in mind-wandering reports (N = 152, n = 1496) from waking well-being and ill-being

LIWC Category	Predictor	*B*	95% CI LL	95% CI UL	*SE*	*t*	*P*	Adjusted *P*
**Positive tone**	**Well-Being**	**0.092**	**0.023**	**0.162**	**0.035**	**2.63**	.010^*^	.038^*^
	Ill-Being	0.014	−0.056	0.084	0.035	0.40	.692	.808
	Age	0.024	−0.042	0.089	0.033	0.72	.472	
	Gender: Man	−0.031	−0.199	0.136	0.085	−0.37	.711	
	Gender: Other	−0.136	−0.441	0.169	0.154	−0.88	.380	
	Country: UK	0.034	−0.090	0.159	0.063	0.55	.584	
**Negative tone**	Well-Being	−0.006	−0.077	0.066	0.036	−0.16	.872	.970
	**Ill-Being**	**0.169**	**0.097**	**0.240**	**0.036**	**4.63**	< .001^***^	< .001^***^
	Age	0.065	−0.002	0.132	0.034	1.91	.058	
	Gender: Man	−0.021	−0.194	0.151	0.087	−0.24	.809	
	Gender: Other	−0.027	−0.341	0.287	0.159	−0.17	.864	
	Country: UK	0.089	−0.039	0.217	0.065	1.38	.169	
Positive emotion	Well-Being	0.068	0.002	0.134	0.033	2.05	.043^*^	.119
	Ill-Being	0.002	−0.064	0.068	0.033	0.06	.951	.970
	Age	0.019	−0.043	0.081	0.031	0.61	.544	
	Gender: Man	−0.063	−0.222	0.095	0.080	−0.79	.429	
	Gender: Other	−0.139	−0.427	0.149	0.146	−0.95	.342	
	Country: UK	0.000	−0.117	0.118	0.059	0.01	.996	
**Negative emotion**	Well-Being	−0.017	−0.091	0.056	0.037	−0.47	.637	.808
	**Ill-Being**	**0.158**	**0.084**	**0.231**	**0.037**	**4.25**	< .001^***^	< .001^***^
	Age	0.059	−0.010	0.128	0.035	1.70	.091	
	Gender: Man	−0.114	−0.290	0.063	0.089	−1.27	.205	
	Gender: Other	−0.026	−0.346	0.295	0.162	−0.16	.875	
	Country: UK	0.089	−0.042	0.219	0.066	1.34	.182	
**Anxiety**	Well-Being	0.011	−0.041	0.064	0.026	0.43	.671	.808
	**Ill-Being**	**0.087**	**0.035**	**0.140**	**0.027**	**3.28**	.001^**^	.012^*^
	Age	0.004	−0.046	0.053	0.025	0.16	.877	
	Gender: Man	−0.043	−0.170	0.083	0.064	−0.68	.500	
	Gender: Other	−0.037	−0.267	0.193	0.116	−0.32	.752	
	Country: UK	0.132	0.038	0.226	0.047	2.78	.006^**^	
**Anger**	Well-Being	0.019	−0.017	0.055	0.018	1.04	.302	.529
	**Ill-Being**	**0.056**	**0.019**	**0.092**	**0.019**	**3.00**	.003^**^	.020^*^
	Age	0.006	−0.028	0.040	0.017	0.37	.712	
	Gender: Man	−0.077	−0.165	0.010	0.044	−1.75	.083	
	Gender: Other	0.010	−0.148	0.169	0.080	0.13	.897	
	Country: UK	0.074	0.009	0.139	0.033	2.26	.025^*^	
**Sadness**	Well-Being	0.009	−0.022	0.040	0.016	0.57	.571	.808
	**Ill-Being**	**0.045**	**0.013**	**0.076**	**0.016**	**2.82**	.006^**^	.026^*^
	Age	0.028	−0.001	0.057	0.015	1.89	.061	
	Gender: Man	−0.031	−0.106	0.044	0.038	−0.82	.412	
	Gender: Other	−0.002	−0.138	0.134	0.069	−0.03	.978	
	Country: UK	0.017	−0.038	0.072	0.028	0.61	.544	

*Note.*  ^*^P < .05, ^**^P < .01, ^***^P < .001. Coefficients marked in bold remained significant after correcting for multiple comparisons with the Benjamini-Hochberg False Discovery Rate (Benjamini and Hochberg 1995). Gender and country were entered using treatment (dummy) coding, with woman as the reference category for gender and Australia as the reference category for country. *N* = number of participants, *n* = number of reports.

**Figure 2:**
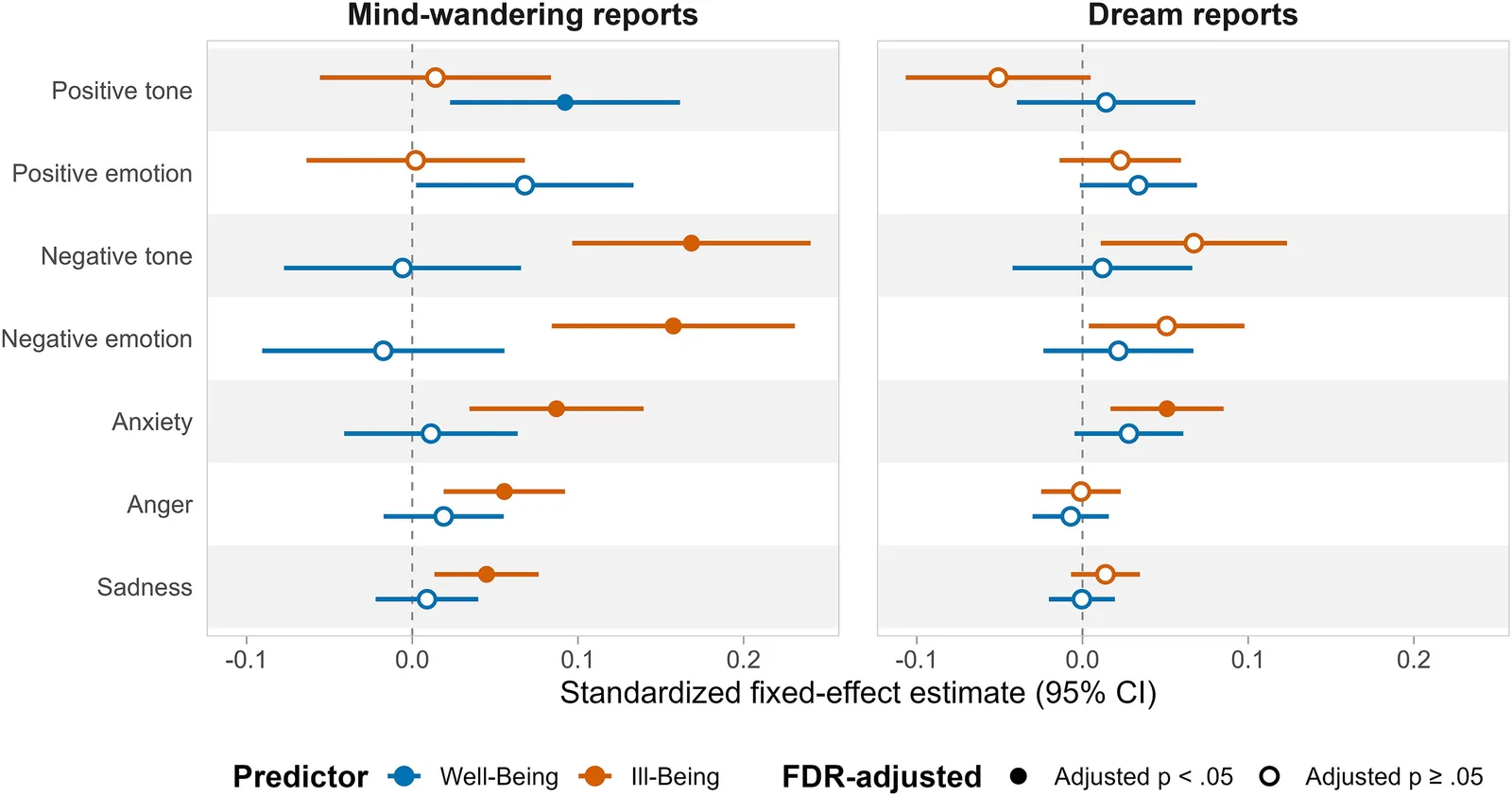
Fixed-effect regression estimates (95% CI) for well-being and ill-being predicting LIWC categories. Each point represents the fixed-effect regression estimate of the predictor (ill-being or well-being) for a specific LIWC category, with error bars indicating 95% confidence intervals for mind-wandering and dream reports. Confidence intervals are unadjusted for multiple comparisons. Filled points indicate effects that remained significant after Benjamini-Hochberg FDR correction across the 28 focal well-being and ill-being tests, hollow points indicate effects that did not. Three effects have confidence intervals excluding zero but did not survive correction. Well-being and ill-being predictors were z-standardized in the regression models, whereas LIWC outcomes were square-root transformed but not standardized. Thus, each estimate represents the expected change in the square-root-transformed LIWC outcome associated with a one-standard-deviation increase in the respective predictor, holding the other variables in the model constant. Positive estimates indicate a positive association between the predictor and the outcome, while negative estimates represent a negative association. The width of the confidence intervals reflects the precision of the estimates, with narrower intervals indicating greater precision.

**Table 4:** Results of linear mixed effects models predicting emotional language use in dream reports (N = 172, n = 1781) from waking well-being and ill-being

LIWC Category	Predictor	*B*	95% CI LL	95% CI UL	*SE*	*t*	*P*	Adjusted *P*
Positive tone	Well-Being	0.014	−0.040	0.068	0.027	0.52	.602	.808
	Ill-Being	−0.051	−0.107	0.005	0.028	−1.80	.074	.172
	Age	−0.021	−0.073	0.031	0.026	−0.81	.421	
	Gender: Man	−0.045	−0.179	0.088	0.067	−0.67	.502	
	Gender: Other	−0.019	−0.254	0.215	0.119	−0.16	.872	
	Country: UK	−0.072	−0.169	0.024	0.049	−1.48	.141	
Negative tone	Well-Being	0.012	−0.042	0.066	0.027	0.44	.662	.808
	Ill-Being	0.067	0.011	0.123	0.028	2.36	.019^*^	.068
	Age	−0.050	−0.102	0.002	0.026	−1.91	.058	
	Gender: Man	0.048	−0.086	0.182	0.068	0.71	.478	
	Gender: Other	0.006	−0.230	0.243	0.120	0.05	.958	
	Country: UK	−0.055	−0.152	0.042	0.049	−1.12	.267	
Positive emotion	Well-Being	0.034	−0.002	0.069	0.018	1.89	.061	.155
	Ill-Being	0.023	−0.014	0.059	0.019	1.23	.221	.412
	Age	0.007	−0.027	0.041	0.017	0.41	.681	
	Gender: Man	−0.002	−0.089	0.086	0.044	−0.04	.971	
	Gender: Other	0.048	−0.107	0.202	0.078	0.61	.544	
	Country: UK	−0.025	−0.088	0.039	0.032	−0.77	.443	
Negative emotion	Well-Being	0.022	−0.024	0.067	0.023	0.95	.346	.570
	Ill-Being	0.051	0.004	0.098	0.024	2.14	.034^*^	.106
	Age	−0.032	−0.075	0.012	0.022	−1.45	.150	
	Gender: Man	0.057	−0.055	0.169	0.057	1.01	.313	
	Gender: Other	0.030	−0.168	0.227	0.100	0.30	.767	
	Country: UK	−0.135	−0.216	−0.054	0.041	−3.30	.001^**^	
**Anxiety**	Well-Being	0.028	−0.005	0.061	0.017	1.68	.094	.203
	**Ill-Being**	**0.051**	**0.017**	**0.085**	**0.017**	**2.95**	.004^**^	.020^*^
	Age	−0.020	−0.051	0.012	0.016	−1.24	.217	
	Gender: Man	0.043	−0.038	0.124	0.041	1.04	.298	
	Gender: Other	−0.010	−0.154	0.134	0.073	−0.14	.890	
	Country: UK	−0.081	−0.140	−0.023	0.030	−2.74	.007^**^	
Anger	Well-Being	−0.007	−0.030	0.016	0.012	−0.61	.542	.808
	Ill-Being	−0.001	−0.025	0.023	0.012	−0.08	.938	.970
	Age	−0.011	−0.033	0.011	0.011	−1.01	.316	
	Gender: Man	−0.000	−0.057	0.056	0.029	−0.01	.995	
	Gender: Other	0.047	−0.055	0.148	0.051	0.91	.365	
	Country: UK	−0.056	−0.098	−0.015	0.021	−2.71	.008^**^	
Sadness	Well-Being	−0.000	−0.020	0.020	0.010	−0.04	.970	.970
	Ill-Being	0.014	−0.007	0.035	0.011	1.32	.190	.380
	Age	−0.006	−0.025	0.013	0.010	−0.65	.517	
	Gender: Man	−0.011	−0.060	0.038	0.025	−0.45	.655	
	Gender: Other	0.009	−0.079	0.096	0.044	0.19	.846	
	Country: UK	−0.013	−0.048	0.023	0.018	−0.69	.489	

*Note.*  ^*^P < .05, ^**^P < .01, ^***^P < .001. Coefficients marked in bold remained significant after correcting for multiple comparisons with the Benjamini-Hochberg False Discovery Rate (Benjamini and Hochberg 1995). Gender and country were entered using treatment (dummy) coding, with woman as the reference category for gender and Australia as the reference category for country. *N* = number of participants, *n* = number of reports.

To assess whether the distributional assumptions of primary models influenced our conclusions, we refitted all models as Tweedie generalized linear mixed models on the untransformed outcomes. All 28 focal tests were in the same direction and 27 of 28 reached the same conclusion after FDR (see Supplementary Table 5). The single exception was the association between well-being and positive tone in mind-wandering reports, which was not significant under the Tweedie models (adjusted *p* = .215, compared with .038 in the primary analysis). The associations between ill-being and negative emotional language use in mind-wandering reports and between ill-being and anxiety words in dream reports were unchanged.

Sensitivity analyses excluding reports containing ˂25 words (50 mind-wandering reports and 125 dream reports were excluded, leaving 1446 and 1656 reports, respectively) yielded a broadly similar pattern of findings (see Supplementary Tables 6 and 7). In mind-wandering reports, the five ill-being associations remained significant after FDR correction. The association between well-being and positive tone no longer survived correction (adjusted *p* = .038 versus .137). In dream reports, the single association between ill-being and anxiety words that survived correction in the primary analyses was attenuated and no longer significant (B = 0.051 versus 0.041; adjusted *p* = .020 versus .082). Thus, the associations between ill-being and negative emotional language use in mind-wandering reports were robust to the exclusion of very short reports, whereas the weaker dream associations were not.

Taken together, the five associations between ill-being and negative emotional language use in mind-wandering reports were significant in the primary models, Tweedie models, and in sensitivity analyses. The association between ill-being and anxiety words in dream reports was also significant in both primary and Tweedie models but attenuated below significance when reports of ˂25 words were excluded. The association between well-being and positive tone in mind-wandering reports was significant only in the primary models and should therefore be interpreted with caution.

### Relationship between individual well-being and ill-being scales and emotional language use in mind-wandering and dream reports

Follow-up models, in which we used the individual well-being and ill-being scales as separate predictors, revealed that in mind-wandering reports each of the three ill-being scales (i.e. negative affect, depression, anxiety) individually predicted the five negative-valence LIWC categories (negative tone, negative emotion, anxiety, anger, and sadness). The only exception was anger, where symptoms of depression did not reach significance (see Supplementary Table 8). Among the three ill-being measures, symptoms of anxiety, and negative affect were the strongest overall predictors. Of the five well-being scales, eudaimonic well-being and peace of mind predicted positive tone words after FDR correction. In dream reports, negative affect (but not symptoms of anxiety or depression) individually predicted the use of anxiety words (see Supplementary Table 9).

### Relationship between LIWC emotional language scores and self-ratings of affect

Additional analyses showed that self-rated affect was associated with corresponding LIWC emotional language scores at both the within- and between-person levels in both report types (all *p*s < .001; see Supplementary Table 10). This provides convergent evidence for an association between emotional language use and self-ratings of affect. Because participants provided a single overall rating of positive and negative affect for each episode, this convergence pertains to global affective tone rather than for discrete emotions.

## Discussion

We investigated whether the emotional language people use to describe spontaneous thoughts and experiences—such as mind-wandering and dreaming—reflects their mental well-being and ill-being. Our findings provide evidence for a link between ill-being and emotional language use and more limited evidence for a link with well-being. In particular, ill-being (characterized by negative affect, anxiety, and depression symptoms) was associated with negative tone as well as the use of negative emotion words, including anxiety, anger, and sadness, in mind-wandering reports. We also found an association between well-being (specifically eudaimonic well-being and peace of mind) and the use of positive tone words, although this association was weaker and did not hold in sensitivity analyses. In dream reports, the association between ill-being and emotional language use was weaker and limited to anxiety words. Although well-being did not predict the use of emotional words in our dream dataset, ill-being predicted the content of both mind-wandering and dream reports.

Overall, measures of self-reported negative affect over the past 2 weeks predicted negative word use across both mind-wandering and dream reports. Additionally, symptoms of anxiety and depression also predicted almost all aspects of negative word use in mind-wandering reports. Replicating previous work on analyses of clinical interviews (Stade et al. 2023), our results showed that depression was linked to the use of sadness words, while anxiety was linked to a broad range of negative emotion words, but especially with anxiety words. These findings align with patterns observed in studies of social media texts where negative emotion words, particularly sadness, were positively associated with self-reported depression and anxiety (Settanni and Marengo 2015; Eichstaedt et al. 2018).

Our findings are also consistent with research on therapy transcripts and simulated telehealth psychiatric intake interviews, where higher depression scores were associated with the use of negative tone, negative emotion, anxiety, and sadness—but not anger—words (Corbin et al. 2023), with sadness words more closely related to measures of depression than anxiety (Sonnenschein et al. 2018). Our observation that depression was associated with all negative emotion word categories except anger echoes earlier findings suggesting that anger word use increases as depression symptoms subside (Lieberman and Goldstein 2006). However, unlike in previous findings (Stade et al. 2023), depression was not significantly associated with lesser use of positive emotion words. Together, these findings support the notion that the emotional language used in mind-wandering and dream reports indeed seems to reflect ill-being.

Our results suggest that reports of spontaneous experiences could be a useful tool for tracking mental ill-being, complementing other types of texts. For example, while social media conveniently provides vast amounts of texts produced by people, it also comes with challenges. Jaidka et al. (2020) observed that socioeconomic, cultural, and regional variations may drive inconsistencies in the utility of such methods for predicting well-being from tweets. In addition, social media posts are subject to biases such as strategic self-presentation, with individuals more likely to share positive sentiments in public (Bazarova et al. 2012). Initial findings also hint toward stronger associations between linguistic markers and measures of ill-being in private (rather than public; Edwards and Holtzman 2017) and informal (rather than formal; Havigerová et al. 2019) settings. Thus, analyzing the content of reports of spontaneous experiences, like mind-wandering and dream reports, which are less susceptible to these biases, may offer more accurate insights into mental health. The asymmetry we observed, with stronger and more robust associations for ill-being than for well-being, is consistent with this literature, in which linguistic markers have generally tracked ill-being more reliably than well-being.

More broadly, the present findings contribute to growing efforts to understand how affective experience varies across different states of consciousness. Research on affect has predominantly focused on active wakefulness, despite the fact that conscious experience extends across states characterized by markedly different relationships to external environment, including mind-wandering and dreaming (Sikka and Gross 2023). Examining affect across these states may help identify which features of affective experience show continuity across waking and sleeping consciousness and which are state-dependent. The present finding that individual differences in ill-being are associated with emotional language not only during mind-wandering but also during dreaming is consistent with some degree of continuity in affective processes across conscious states, while differences in the strength and pattern of these associations point to potential state-dependent variation. Related work has similarly linked negative affect during both mind-wandering and dreaming to greater worry during wakefulness (Sikka et al. 2023b), whereas dream fear has been associated with greater affect reactivity (Lin et al. 2026). Examining how diurnal variation in affective processes (Bu et al. 2025) relates to affect during sleep may thus help elucidate a more comprehensive account of how emotional experience is organized across the day-night cycle and across different states of consciousness.

Theoretically, our findings align with the dynamic framework of thought (Christoff et al. 2016), suggesting that mind-wandering and dreaming are related but distinct states of consciousness, both reflecting affective states across the day-night cycle. The stronger association between ill-being and mind-wandering reports, as compared to dream reports, suggests that mind-wandering is more closely tied to real-time emotional and cognitive processes, particularly anxiety and negative emotions. In contrast, dreaming may reflect more enduring emotional themes, potentially shaped by memory consolidation processes during sleep that integrate and stabilize emotional experiences over time. Thus, while mind-wandering seems to reflect immediate emotional states, dreaming might instead engage broader, long-term emotional processing.

It is possible, however, that it is not the experiences per se that are different (i.e. more negative), but rather the way individuals describe them. For example, more anxious individuals may simply use more negative words when describing their (both daytime and night-time) experiences, compared to less anxious individuals. Indeed, trait-like individual differences have been suggested to underlie continuity between daytime and nighttime experiences (Sikka et al. 2023b). Nevertheless, the language used in these reports, whether or not the experiences themselves are inherently more negative, appears to be an important indicator of ill-being.

Future studies could compare dream and mind-wandering reports with social media posts as predictors of mental health and examine other aspects of language that have been tied to variations in well-being (e.g. words related to loneliness, hostility, and I-language). Additionally, exploring the relationship between emotional language use and other markers of emotional well-being (e.g. alexithymia; Preece et al. 2017) and emotion regulation may provide further insight into how language reflects mental health.

Our findings should be considered in light of several limitations. The observed association between emotional language use and ill-being, and more modestly well-being, may partly reflect the context of data collection during the early stages of the COVID-19 pandemic, when levels of ill-being were globally elevated (Gallup 2021). Indeed, we did find that country predicted the use of negative emotion, anxiety, and anger words, albeit in opposite directions across the two report types. It would be interesting to further explore the relationship between emotional language use and mental health across a variety of contexts, countries, and cultures.

A further reason why ill-being predicted emotional language use more strongly than well-being may be the human tendency to express negative experiences relatively more explicitly and positive experiences more implicitly (Sikka et al. 2014, 2017), combined with the context-blindness of LIWC. Since LIWC plainly measures word frequencies regardless of context (e.g. negation, sarcasm, and metaphor), it cannot distinguish between the meaning of ‘I am sad’ and ‘I am definitely not sad’, or capture nuances in the meanings of ambiguous words such as ‘mad’ (e.g. ‘I feel mad at them’ and ‘they were madly in love’) (Pennebaker Conglomerates, n. d.). Thus, it may be that these constraints cause LIWC to bypass the more implicitly expressed positive emotions. This insensitivity to context constitutes a significant limitation inherent in bag-of-words methods such as LIWC, which highlights the need for more nuanced, context-sensitive tools in research using natural language processing.

The strong negativity bias in LIWC vocabularies may contribute to the same asymmetry. Since LIWC negative vocabularies contain significantly more words than the positive vocabularies do (e.g. 337 words in the ‘positive emotion’ category *versus* 618 words in the ‘negative emotion’ category), LIWC may capture negative content in the reports more effectively than it does positive content. This may also help explain why the association between well-being and positive tone was the least robust of our findings.

Similarly, the presence of modifiers in tone vocabularies (such as ‘too much’ in the negative tone vocabulary) may lead to ambiguous or erroneous sentiment characterization in some reports. For example, in the phrase ‘too much joy’, LIWC-22 would count ‘too much’ toward negative tone and ‘joy’ toward positive tone, without evaluating the meaning of the phrase as a whole. Thus, the resulting scores reflect the dictionary classifications of the constituent words or expressions rather than a context-sensitive interpretation of the phrase. The issue may be particularly relevant for shorter reports, in which individual dictionary matches have a greater influence on percentage-based LIWC scores. Consistent with this, excluding reports of ˂25 words attenuated the two weakest associations in our data, while leaving the associations between ill-being and negative emotional language use in mind-wandering reports unchanged. It should further be noted that, unlike the tone vocabularies, the emotion vocabularies used in the present study do not contain such modifiers and are therefore not affected by this particular issue. More generally, this illustrates a limitation intrinsic to closed-vocabulary approaches: valence is assigned at the level of the word rather than the phrase, so negation, intensification, and irony may not be adequately captured.

Over time, new linguistic approaches have evolved that extend and complement LIWC by automatically extracting content from large corpora of experience reports in increasingly potent and nuanced ways (for a review, see Elce et al. 2021). For example, Fogli et al. (2020) presented a tool for automatically scoring enormous sets of dream reports in line with a well-established manual coding system. This new method quickly extracts detailed insights about characters, interactions, and emotions, i.e*.* three highly informative dimensions of dream narratives, from large datasets, and allowed the researchers to analyze 24 000 dream reports to explore broader questions and find support for the continuity hypothesis. Moreover, Das et al. (2025) presented an unsupervised deep-learning framework that discovers dream themes and patterns in large-scale data without the use of scoring categories or pre-existing structures. The method aims to reflect the spontaneous nature of dreams more closely and with higher ecological validity and nuance than traditional, structured methods can. As such, it can be used to augment and balance traditional methods. In their study, analysis of over 44 000 dream reports yielded 217 topics grouped into 22 overarching themes. While most of these overlapped with traditional scoring categories, some new, previously undetected themes also emerged.

Broadly, studies using machine learning methods, including large language models (Demszky et al. 2023), offer promising alternatives to LIWC by capturing contextual nuances and reducing vocabulary-driven biases in emotion detection. While LIWC is a well-established tool in psychological research, recent advancements in AI technologies provide opportunities for complex lexical analysis that may increasingly be used to augment LIWC. These methods should be applied and compared in follow-up studies, in ways that respect known issues (such as inherent biases in the training data).

In this study, participants were asked to intentionally engage in mind-wandering, a process that differs from the unintentional mind-wandering often studied outside the lab, where participants are prompted randomly throughout the day (Smallwood and Schooler 2015). This approach, commonly used in resting-state fMRI studies (Gonzalez-Castillo et al. 2021), was chosen to ensure consistency with the dream reporting procedure. However, spontaneous thoughts emerging during intentional mind-wandering may differ from those arising unprompted. Although immediate sampling throughout the day might provide different insights (Gross et al. 2021), the procedure used in this study ensured consistent reporting across both mind-wandering and dream experiences.

Relatedly, although participants were given instructions regarding when to perform the mind-wandering task and report their dreams, no practical constraints prevented them from performing the tasks at times other than those specified (i.e*.* dream reporting in the morning and mind-wandering in the evening). Time-of-day and circadian factors may influence both reported well-being and the content of conscious experiences (Bu et al. 2025). Allowing participants some flexibility in scheduling the tasks was a deliberate choice. It accommodated individual chronotypes and the practical demands of home-based daily participation over 2 weeks, particularly given that data were collected during COVID-19 restrictions, when many participants were managing work, caregiving, and other responsibilities at home. Stricter enforcement would likely have reduced compliance and increased attrition. However, this flexibility leaves open the possibility that task and reporting times varied both within and between participants. Future studies could reduce this uncertainty by implementing more restricted reporting windows and recording the timing of both task completion and reporting more precisely.

In this study, timestamps indicating when the reports were submitted were automatically collected. However, these timestamps may not reflect when the experience was initially reported because participants were allowed to first write down their reports on paper or elsewhere before transferring them to the online reporting system. Thus, the interval between the initial recording of the experience and its online submission could vary. For this reason, we did not further analyze reporting times.

A few methodological challenges related to dream recall and reporting should be noted. Dream reports necessarily constitute a selective and potentially incomplete representation of dream experiences because they depend on what is attended to, encoded, retained, and subsequently recalled upon awakening. Dream memories may fade rapidly following awakening and may be further affected by interference and by the delay between the dream experience and its report (Parke and Horton 2009; Sikka 2020). In the present study, participants were instructed to take notes of their dreams immediately upon awakening, which should have reduced such memory effects. However, because the initial notes could be transferred into the online reporting system later, some dream content may nevertheless have been forgotten or modified during this process. Importantly, the dreams recalled upon spontaneous morning awakening are unlikely to constitute a random sample of all dream experiences occurring during the night. The most recent and affectively salient (especially negative) dream experiences may be particularly likely to be remembered in the morning, with recency being an especially important factor (Domhoff 2005).

Moreover, later-night rapid-eye movement (REM) sleep dreams tend to be more emotional than earlier-night REM dreams (Sikka et al. 2018a). Thus, the emotional characteristics of dreams may themselves influence their likelihood of being recalled. Dream recall and reporting are also subject to substantial individual differences, including differences in attention to and interest in dreams, participant’s introspection and reporting skills, mind-wandering tendency, and trait differences in sleep patterns (Kahan and Claudatos 2016; Sikka et al. 2017; Elce et al. 2025). Taken together, dream reports analyzed here should not be regarded as a representative sample of all dream experiences occurring across the night.

It should further be noted that all reports were collected in written form. Compared with transcribed oral reports, written reports may involve a greater degree of reflective reconstruction and less immediate expression (Casagrande and Cortini 2008). Several features of our procedure were designed to minimize such reconstruction: participants were instructed to write their reports immediately after the mind-wandering period and immediately upon awakening, not to revise their reports with later additions or conclusions or to make the experience more logical or complete than remembered, and to record any waking commentary in brackets so that it could be removed prior to analysis. Importantly, because both report types were collected using the same modality, reporting modality cannot account for the differences observed between mind-wandering and dream reports. Nevertheless, it is possible that oral rather than written reports would have yielded different results. Future studies could therefore collect reports in both oral and written form and directly compare the resulting findings.

Moreover, although gender was controlled for, the generalizability of the results is limited by the gender imbalance in the sample, as four out of five participants were women. Juxtaposing the language of women and men, previous work from large-scale studies suggests that text produced by women tends to contain more references to internal processes, social relationships, friendly interactions, emotions (especially positive), as well as more negations, while men’s language contains more associations to external and impersonal objects and processes, negative emotions, and tends to be colder and more hostile (Newman et al. 2008; Park et al. 2016; Fogli et al. 2020; Eichstaedt et al. 2021). Given these patterns, it may be that a sample featuring predominantly men would produce different results, such as less emotionality overall—echoing the commonly reported higher rates of alexithymia in men (Preece et al. 2022)—as well as a tendency toward more negative emotion words in particular. To clarify the potential impact of these tendencies on the relationship between emotional language use in reports of spontaneous experiences and mental health, future studies should aim toward greater gender balance.

Further relating to the sample, participants were not required to be native speakers of English and their first language or English proficiency was not recorded. The study was advertised and participants were recruited in Australia and the United Kingdom, both of which are home to populations with high levels of native or high-proficiency English speakers (Australian Bureau of Statistics 2022; Office for National Statistics 2022). During data preparation, all reports were read in full and none were excluded on the grounds of English language proficiency or readability. Since participants were required to provide detailed free-form reports in English daily over a 2-week period, this requirement may have introduced a degree of self-selection favoring individuals comfortable with written English.

Finally, given the correlational nature of the study, no causal relationships are revealed from its results. However, future studies involving longitudinal designs may shed light on the direction of causality, elucidating whether, e.g*.* ill-being causes individuals to express themselves more negatively, or whether instead negative language use can contribute to ill-being (e.g*.* by providing conceptual frames that shape how experiences are constructed; Lindquist et al. 2015). Assessing well-being and ill-being over extended periods may provide insights into how changes in language use may correspond to changes in well-being and ill-being (e.g*.* among at-risk students or in therapeutic contexts). Longitudinal designs may also be beneficial due to their capacity to consistently capture experiences over time. While in this study, participants only rated their waking well-being and ill-being on one occasion (i.e. before commencing the 2 weeks of dream diaries and/or the mind-wandering task), longitudinal and more time-sensitive approaches (e.g*.* taking into account what time of the day or days of the week participants fill out the questionnaires) may reflect participants’ waking emotional experiences more accurately (Bu et al. 2025). This may especially be the case as many women of reproductive age (who make up a large portion of the participants in psychological studies) experience significant fluctuations in mood throughout the month due to hormonal shifts (Dubol et al. 2021; Handy et al. 2022).

## Conclusions

In conclusion, our study highlights the potential of analyzing emotional language in mind-wandering and dream reports as a tool for understanding mental health, particularly mental ill-being. Although mind-wandering reports appear more sensitive to these links, both types of spontaneous experience reports offer valuable insights that could inform the development of more effective prognostic and diagnostic tools in clinical settings. Longitudinal research is needed to clarify the causal relationships between emotional language use and mental health.

## Supplementary Material

NCONSC-2026-060_R1_Supplementary_File_20261022_CLEAN_niag075

## Data Availability

Deidentified data used in this study can be found at the project’s Open Science Framework page (Study 4): https://osf.io/su7mn/.

## Code availability

Analysis scripts used in this study can be found at the project’s Open Science Framework page: https://osf.io/su7mn/ (Study 4).
