# Supplementary material for: The words of our inner worlds: emotional language use in mind-wandering and dream reports reflects mental ill-being
Source: Neurosci Conscious. 2026 Oct 31;2026(1):niag075. doi: 10.1093/nc/niag075 (PMC13755700; doi:10.1093/nc/niag075)
Supplement: Supplementary file 1 [file nconsc-2026-060_r1_supplementary_file_20261022_clean_niag075.docx]

**Supplementary Information for:**

**The Words of Our Inner Worlds: Emotional Language Use in Mind-Wandering and Dream Reports Reflects Mental Ill-Being**

Nanna Strid ^a, b^, Jarno Tuominen ^a^, Ryan Bernstein ^c^, Manuela Kirberg ^d, e^, Katja Valli ^a, b^, Jennifer Windt ^d, e^, Tristan Bekinschtein ^f^, Valdas Noreika ^g^, Antti Revonsuo ^a, b^, and Pilleriin Sikka ^a, b, c, h^.*

^a^ Department of Psychology and Speech-Language Pathology and Turku Brain and Mind Center, University of Turku, Turku, Finland

^b^ Department of Cognitive Neuroscience and Philosophy, University of Skövde, Skövde, Sweden

^c^ Department of Psychology, Stanford University, Stanford, CA, USA

^d^ Department of Philosophy, Monash University, Clayton, VIC, Australia

^e^ Monash Centre for Consciousness and Contemplative Studies, Monash University, Clayton, VIC, Australia

^f^ Consciousness and Cognition Lab, Department of Psychology, University of Cambridge, Cambridge, United Kingdom

^g^ Department of Biological and Experimental Psychology, School of Biological and Behavioural Sciences, Queen Mary University of London, London, United Kingdom

^h^ Department of Anesthesiology, Perioperative and Pain Medicine, School of Medicine, Stanford University, Stanford, CA, USA

***Correspondence concerning this article should be addressed to:**

Pilleriin Sikka, Department of Anesthesiology, Perioperative and Pain Medicine, Stanford University, 94305 CA, USA, [sikka@stanford.edu](mailto:sikka@stanford.edu)

**Correlations Between Well-Being Scales, Ill-Being Scales, and Emotional Language Use in Mind-Wandering and Dream Reports**

As expected, well-being scales correlated positively with each other and negatively with ill-being scales (see Table S2). Tables S3 and S4 present correlations between all well-being and ill-being scales and LIWC variables. With regard to mind-wandering reports, positive tone correlated positively with measures of eudaimonic well-being, peace of mind, and the well-being composite; positive emotion words with all well-being scales and the well-being composite. Negative tone and negative emotion words were negatively correlated with all well-being scales and the well-being composite; anxiety words with measures of life satisfaction, peace of mind, and the well-being composite. Additionally, positive tone correlated negatively with measures of negative affect and the ill-being composite; positive emotion words with symptoms of depression, negative affect, and the ill-being composite, but not with anxiety. Both negative tone and negative emotion words correlated positively with all ill-being measures. Anxiety, anger, and sadness words correlated positively with symptoms of anxiety, negative affect, and the ill-being composite, but not with symptoms of depression.

In dream reports, the correlations were generally weaker. Positive tone was positively associated with self-rated positive affect and the well-being composite; positive emotion with measures of life satisfaction and positive affect. Negative tone correlated negatively with measures of positive affect and peace of mind, while both negative emotion and anger words were negatively associated with self-rated positive affect. Moreover, positive tone was negatively correlated with depression symptoms, but not with anxiety, negative affect, or the ill-being composite. Conversely, negative tone as well as negative emotion and anxiety words correlated positively with all ill-being scales; sadness words with self-rated negative affect and the ill-being composite, but neither with anxiety nor depression symptoms. Anger words did not correlate with any of the ill-being scales.

**Supplementary Table 1**

*Principal Component Analysis: Component Loadings*

| Scale | PC1 | PC2 | PC3 | PC4 | PC5 | PC6 | PC7 | PC8 |
| --- | --- | --- | --- | --- | --- | --- | --- | --- |
| SWLS | -0.36 | 0.31 | -0.27 | 0.57 | -0.29 | 0.29 | 0.40 | 0.23 |
| BBQ | -0.34 | 0.31 | -0.46 | -0.28 | 0.69 | 0.12 | -0.02 | -0.04 |
| PANAS-PA | -0.35 | 0.18 | 0.67 | -0.37 | 0.06 | 0.05 | 0.48 | 0.12 |
| PWB | -0.36 | 0.37 | 0.19 | 0.31 | -0.00 | -0.66 | -0.29 | -0.28 |
| PoM-5 | -0.39 | 0.10 | -0.20 | -0.51 | -0.61 | 0.13 | -0.38 | 0.02 |
| PHQ-9 | 0.37 | 0.31 | -0.36 | -0.30 | -0.24 | -0.43 | 0.53 | -0.16 |
| GAD-7 | 0.34 | 0.50 | 0.11 | -0.03 | 0.04 | -0.07 | -0.30 | 0.72 |
| PANAS-NA | 0.31 | 0.53 | 0.21 | 0.05 | -0.04 | 0.50 | -0.14 | -0.55 |

*Note.* SWLS = Satisfaction with Life Scale. BBQ = Brunnsviken Brief Quality of Life Scale (BBQ). PANAS = Positive Affect Negative Affect Schedule. PA = positive affect. NA = negative affect. PWB = Ryff's Scales of Psychological Well-Being. PoM-5 = Peace of Mind Scale. PHQ-9 = Depression scale from Patient Health Questionnaire. GAD-7 = Generalized Anxiety Disorder Scale.

**Supplementary Table 2**

*Correlations (Spearman's* ρ*) Between Well- and Ill-Being Scales (N = 176)*

| Scale | SWLS | BBQ | PANAS-  PA | PWB | PoM-5 | WB-C | PHQ-9 | GAD-7 | PANAS-  NA |
| --- | --- | --- | --- | --- | --- | --- | --- | --- | --- |
| SWLS |  |  |  |  |  |  |  |  |  |
| BBQ | .56*** |  |  |  |  |  |  |  |  |
| PANAS-PA | .39*** | .39*** |  |  |  |  |  |  |  |
| PWB | .63*** | .49*** | .55*** |  |  |  |  |  |  |
| PoM-5 | .55*** | .54*** | .50*** | .50*** |  |  |  |  |  |
| WB-C | .80*** | .75*** | .72*** | .80*** | .78*** |  |  |  |  |
| PHQ-9 | -.33*** | -.26*** | -.48*** | -.39*** | -.40*** | -.47*** |  |  |  |
| GAD-7 | -.30*** | -.28*** | -.31*** | -.22** | -.47*** | -.40*** | .64*** |  |  |
| PANAS-NA | -.22** | -.24** | -.24** | -.20** | -.41*** | -.34*** | .61*** | .76*** |  |
| IB-C | -.32*** | -.29*** | -.39*** | -.31*** | -.49*** | -.45*** | .84*** | .90*** | .90*** |

*Note.* ****p* < .001. *N* = number of participants. SWLS = Satisfaction with Life Scale. BBQ = Brunnsviken Brief Quality of Life Scale (BBQ). PANAS = Positive Affect Negative Affect Schedule. PA = positive affect. NA = negative affect. PWB = Ryff's Scales of Psychological Well-Being. PoM-5 = Peace of Mind Scale. PHQ-9 = Depression scale from Patient Health Questionnaire. GAD-7 = Generalized Anxiety Disorder Scale. WB-C = well-being composite, IB-C = ill-being composite. Includes 172 participants with dream logs (of whom 148 provided mind-wandering logs) plus 4 participants who provided mind-wandering logs but not dream logs (152 participants with mind-wandering logs in total).

**Supplementary Table 3**

*Correlations (Spearman's* ρ*) Between Well- and Ill-Being Scales and Emotional Language Use (LIWC Categories) in Mind-Wandering Reports (N = 1496)*

| Scale | Positive  Tone | Negative  Tone | Positive  Emotion | Negative  Emotion | Anxiety | Anger | Sadness |
| --- | --- | --- | --- | --- | --- | --- | --- |
| SWLS | .03 | -.10*** | .09*** | -.10*** | -.10*** | .01 | -.00 |
| BBQ | .02 | -.10*** | .06* | -.12*** | -.04 | .00 | -.00 |
| PANAS-PA | .04 | -.09*** | .05* | -.08** | -.02 | .02 | -.00 |
| PWB | .11*** | -.06* | .10*** | -.08** | -.03 | .01 | -.00 |
| PoM-5 | .07** | -.11*** | .08** | -.09*** | -.05* | -.02 | -.00 |
| WB-C | .06* | -.10*** | .08** | -.11*** | -.06* | .01 | -.00 |
| PHQ-9 | -.04 | .13*** | -.06* | .13*** | .05 | .04 | .04 |
| GAD-7 | -.03 | .20*** | -.02 | .22*** | .16*** | .10*** | .05* |
| PANAS-NA | -.09*** | .17*** | -.05* | .19*** | .14*** | .09*** | .07** |
| IB-C | -.07** | .19*** | -.06* | .21*** | .14*** | .08** | .06* |

*Note.* **p* < .05, ** *p* < .01, ****p* < .001. IB = ill-being, WB = well-being. LIWC = Linguistic Inquiry and Word Count. *N* = number of participants, *n* = number of reports. SWLS = Satisfaction with Life Scale. BBQ = Brunnsviken Brief Quality of Life Scale (BBQ). PANAS = Positive Affect Negative Affect Schedule. PA = positive affect. NA = negative affect. PWB = Ryff's Scales of Psychological Well-Being. PoM-5 = Peace of Mind Scale. PHQ-9 = Depression scale from Patient Health Questionnaire. GAD-7 = Generalized Anxiety Disorder Scale. WB-C = well-being composite, IB-C = ill-being composite.

**Supplementary Table 4**

*Correlations (Spearman's* ρ*) Between Well- and Ill-Being Scales and Emotional Language Use (LIWC Categories) in Dream Reports (N = 1781)*

| Scale | Positive  Tone | Negative  Tone | Positive  Emotion | Negative  Emotion | Anxiety | Anger | Sadness |
| --- | --- | --- | --- | --- | --- | --- | --- |
| SWLS | .04 | -.02 | .06* | .04 | .04 | .02 | .02 |
| BBQ | .04 | .03 | .03 | .05 | .04 | .01 | .02 |
| PANAS-PA | .06* | -.10*** | .05* | -.07** | -.04 | -.06* | -.02 |
| PWB | .02 | -.03 | .01 | -.02 | .00 | -.03 | -.03 |
| PoM-5 | .04 | -.06** | .01 | -.01 | -.02 | -.02 | -.05* |
| WB-C | .05* | -.04 | .04 | -.01 | -.00 | -.02 | -.02 |
| PHQ-9 | -.05* | .09*** | .02 | .07** | .05* | .04 | .03 |
| GAD-7 | -.04 | .09*** | .02 | .06** | .07** | .03 | .04 |
| PANAS-NA | -.01 | .12*** | .05 | .10*** | .11*** | .04 | .07** |
| IB-C | -.04 | .12*** | .03 | .09*** | .09*** | .05 | .05* |

*Note.* **p* < .05, ** *p* < .01, ****p* < .001. IB = ill-being, WB = well-being. LIWC = Linguistic Inquiry and Word Count. *N* = number of participants, *n* = number of reports. SWLS = Satisfaction with Life Scale. BBQ = Brunnsviken Brief Quality of Life Scale (BBQ). PANAS = Positive Affect Negative Affect Schedule. PA = positive affect. NA = negative affect. PWB = Ryff's Scales of Psychological Well-Being. PoM-5 = Peace of Mind Scale. PHQ-9 = Depression scale from Patient Health Questionnaire. GAD-7 = Generalized Anxiety Disorder Scale. WB-C = well-being composite, IB-C = ill-being composite.

**Supplementary Table 5**

*Comparison of Primary Linear Mixed-Effects Models and Tweedie Generalized Linear Mixed Models Predicting Emotional Language Use from Waking Well-Being and Ill-Being in Mind-Wandering Reports (N = 152, n = 1496) and in Dream Reports (N = 172, n = 1781)*

| Type of Report | Outcome | Predictor | B  (sqrt-LMM) | Adjusted p (sqrt-LMM) | B  (Tweedie) | Adjusted p  (Tweedie) | Same direction | Same  conclusion |
| --- | --- | --- | --- | --- | --- | --- | --- | --- |
| Mind-wandering | Positive tone | Well-Being | 0.092 | .038 | 0.059 | .215 | Yes | No |
|  | Positive tone | Ill-Being | 0.014 | .808 | 0.003 | .996 | Yes | Yes |
|  | Negative tone | Well-Being | -0.006 | .970 | -0.046 | .552 | Yes | Yes |
|  | Negative tone | Ill-Being | 0.169 | < .001 | 0.185 | .002 | Yes | Yes |
|  | Positive emotion | Well-Being | 0.068 | .119 | 0.101 | .215 | Yes | Yes |
|  | Positive emotion | Ill-Being | 0.002 | .970 | 0.022 | .857 | Yes | Yes |
|  | Negative emotion | Well-Being | -0.017 | .808 | -0.064 | .530 | Yes | Yes |
|  | Negative emotion | Ill-Being | 0.158 | < .001 | 0.225 | .003 | Yes | Yes |
|  | Anxiety | Well-Being | 0.011 | .808 | 0.003 | .996 | Yes | Yes |
|  | Anxiety | Ill-Being | 0.087 | .012 | 0.307 | .023 | Yes | Yes |
|  | Anger | Well-Being | 0.019 | .529 | 0.034 | .857 | Yes | Yes |
|  | Anger | Ill-Being | 0.056 | .020 | 0.260 | .043 | Yes | Yes |
|  | Sadness | Well-Being | 0.009 | .808 | 0.000 | .996 | Yes | Yes |
|  | Sadness | Ill-Being | 0.045 | .026 | 0.277 | .043 | Yes | Yes |
| Dreams | Positive tone | Well-Being | 0.014 | .808 | 0.014 | .857 | Yes | Yes |
|  | Positive tone | Ill-Being | -0.051 | .172 | -0.069 | .215 | Yes | Yes |
|  | Negative tone | Well-Being | 0.012 | .808 | 0.015 | .857 | Yes | Yes |
|  | Negative tone | Ill-Being | 0.067 | .068 | 0.107 | .061 | Yes | Yes |
|  | Positive emotion | Well-Being | 0.034 | .155 | 0.133 | .136 | Yes | Yes |
|  | Positive emotion | Ill-Being | 0.023 | .412 | 0.063 | .560 | Yes | Yes |
|  | Negative emotion | Well-Being | 0.022 | .570 | 0.036 | .763 | Yes | Yes |
|  | Negative emotion | Ill-Being | 0.051 | .106 | 0.121 | .120 | Yes | Yes |
|  | Anxiety | Well-Being | 0.028 | .203 | 0.139 | .318 | Yes | Yes |
|  | Anxiety | Ill-Being | 0.051 | .020 | 0.259 | .043 | Yes | Yes |
|  | Anger | Well-Being | -0.007 | .808 | -0.092 | .560 | Yes | Yes |
|  | Anger | Ill-Being | -0.001 | .970 | -0.008 | .996 | Yes | Yes |
|  | Sadness | Well-Being | -0.000 | .970 | -0.045 | .857 | Yes | Yes |
|  | Sadness | Ill-Being | 0.014 | .380 | 0.140 | .506 | Yes | Yes |

*Note*. Both model families included the same fixed effects (well-being, ill-being, age, gender, and country) and a participant-specific random intercept. LMM = linear mixed-effects model fitted to square-root-transformed LIWC outcomes. Tweedie = Tweedie generalized linear mixed model fitted to untransformed LIWC outcomes with a log link. Because the two model families are fitted to different response scales, the coefficients are not comparable in magnitude and should be compared only with respect to direction and statistical significance. Adjusted *p* values are Benjamini–Hochberg corrections (Benjamini & Hochberg, 1995). "Same direction" indicates that the estimate had the same sign in both model families; "same conclusion" indicates that the effect was either significant in both or non-significant in both after correction. All 28 effects were in the same direction, and 27 of 28 reached the same conclusion. The exception was the association between well-being and positive tone in mind-wandering reports, which was significant under the LMM (adjusted *p* = .038) but not under the Tweedie model (adjusted *p* = .215). LIWC = Linguistic Inquiry and Word Count. *N* = number of participants, *n* = number of reports.

**Supplementary Table 6**

*Results of Linear Mixed Effects Models Predicting Emotional Language Use in Mind-Wandering Reports (N =152, n = 1446), Restricted to Reports Containing at Least 25 Words*

| Outcome | Predictor | *B* | 95% CI LL | 95% CI UL | *SE* | *t* | *p* | Adjusted  *p* |
| --- | --- | --- | --- | --- | --- | --- | --- | --- |
| Positive tone | Well-Being | 0.068 | 0.001 | 0.135 | 0.034 | 2.01 | .047* | .137 |
|  | Ill-Being | -0.008 | -0.075 | 0.060 | 0.034 | -0.23 | .822 | .932 |
|  | Age | 0.029 | -0.035 | 0.092 | 0.032 | 0.89 | .376 |  |
|  | Gender: Man | -0.024 | -0.185 | 0.138 | 0.082 | -0.29 | .770 |  |
|  | Gender: Other | -0.085 | -0.379 | 0.210 | 0.149 | -0.57 | .570 |  |
|  | Country: UK | 0.024 | -0.096 | 0.145 | 0.061 | 0.40 | .690 |  |
| **Negative tone** | Well-Being | 0.000 | -0.072 | 0.073 | 0.037 | 0.01 | .992 | .992 |
|  | **Ill-Being** | **0.172** | **0.099** | **0.246** | **0.037** | **4.65** | **< .001***** | **< .001***** |
|  | Age | 0.061 | -0.008 | 0.130 | 0.035 | 1.74 | .085 |  |
|  | Gender: Man | -0.048 | -0.223 | 0.128 | 0.089 | -0.54 | .591 |  |
|  | Gender: Other | -0.032 | -0.352 | 0.288 | 0.161 | -0.20 | .843 |  |
|  | Country: UK | 0.079 | -0.052 | 0.210 | 0.066 | 1.20 | .233 |  |
| Positive emotion | Well-Being | 0.061 | -0.004 | 0.126 | 0.033 | 1.85 | .067 | .171 |
|  | Ill-Being | -0.006 | -0.072 | 0.060 | 0.033 | -0.17 | .865 | .932 |
|  | Age | 0.024 | -0.038 | 0.086 | 0.031 | 0.76 | .447 |  |
|  | Gender: Man | -0.060 | -0.218 | 0.098 | 0.080 | -0.75 | .454 |  |
|  | Gender: Other | -0.127 | -0.414 | 0.161 | 0.145 | -0.87 | .386 |  |
|  | Country: UK | -0.007 | -0.124 | 0.111 | 0.059 | -0.12 | .908 |  |
| **Negative emotion** | Well-Being | -0.015 | -0.090 | 0.060 | 0.038 | -0.38 | .702 | .894 |
|  | **Ill-Being** | **0.161** | **0.085** | **0.236** | **0.038** | **4.19** | **< .001***** | **< .001***** |
|  | Age | 0.058 | -0.013 | 0.130 | 0.036 | 1.61 | .109 |  |
|  | Gender: Man | -0.120 | -0.302 | 0.061 | 0.092 | -1.31 | .193 |  |
|  | Gender: Other | -0.032 | -0.363 | 0.298 | 0.167 | -0.19 | .846 |  |
|  | Country: UK | 0.078 | -0.057 | 0.213 | 0.068 | 1.14 | .256 |  |
| **Anxiety** | Well-Being | 0.016 | -0.036 | 0.069 | 0.027 | 0.61 | .543 | .760 |
|  | **Ill-Being** | **0.087** | **0.034** | **0.140** | **0.027** | **3.25** | **.001**** | **.014*** |
|  | Age | 0.003 | -0.047 | 0.053 | 0.025 | 0.13 | .899 |  |
|  | Gender: Man | -0.055 | -0.182 | 0.072 | 0.064 | -0.86 | .392 |  |
|  | Gender: Other | -0.025 | -0.257 | 0.206 | 0.117 | -0.22 | .829 |  |
|  | Country: UK | 0.119 | 0.024 | 0.214 | 0.048 | 2.49 | .014* |  |
| **Anger** | Well-Being | 0.017 | -0.019 | 0.052 | 0.018 | 0.94 | .351 | .578 |
|  | **Ill-Being** | **0.052** | **0.016** | **0.088** | **0.018** | **2.85** | **.005**** | **.035*** |
|  | Age | 0.003 | -0.031 | 0.036 | 0.017 | 0.17 | .868 |  |
|  | Gender: Man | -0.073 | -0.159 | 0.012 | 0.043 | -1.70 | .092 |  |
|  | Gender: Other | 0.014 | -0.141 | 0.170 | 0.079 | 0.18 | .854 |  |
|  | Country: UK | 0.072 | 0.009 | 0.136 | 0.032 | 2.25 | .026* |  |
| **Sadness** | Well-Being | 0.005 | -0.027 | 0.037 | 0.016 | 0.32 | .750 | .913 |
|  | **Ill-Being** | **0.044** | **0.011** | **0.076** | **0.016** | **2.68** | **.008**** | **.047*** |
|  | Age | 0.030 | -0.000 | 0.059 | 0.015 | 1.96 | .053 |  |
|  | Gender: Man | -0.034 | -0.110 | 0.042 | 0.039 | -0.88 | .380 |  |
|  | Gender: Other | -0.007 | -0.147 | 0.133 | 0.071 | -0.10 | .921 |  |
|  | Country: UK | 0.012 | -0.045 | 0.069 | 0.029 | 0.41 | .684 |  |

*Note.* **p* < .05, ** *p* < .01, ****p* < .001. LIWC = Linguistic Inquiry and Word Count. A sensitivity analysis was performed in which reports containing less than 25 words were excluded. In total, 50 mind-wandering reports containing <25 words were removed and 1446 retained. Coefficients marked in bold remained significant after correcting for multiple comparisons with the Benjamini-Hochberg False Discovery Rate (Benjamini & Hochberg, 1995). Gender and country were entered using treatment (dummy) coding, with woman as the reference category for gender and Australia as the reference category for country. *N* = number of participants, *n* = number of reports.

**Supplementary Table 7**

*Results of Linear Mixed Effects Models Predicting Emotional Language Use in Dream Reports (N = 172, n = 1656), Restricted to Reports Containing at Least 25 Words*

| Outcome | Predictor | *B* | 95% CI LL | 95% CI UL | *SE* | *t* | *p* | Adjusted *p* |
| --- | --- | --- | --- | --- | --- | --- | --- | --- |
| Positive tone | Well-Being | 0.028 | -0.020 | 0.075 | 0.024 | 1.14 | .256 | .511 |
|  | Ill-Being | -0.024 | -0.074 | 0.026 | 0.025 | -0.96 | .339 | .578 |
|  | Age | 0.002 | -0.044 | 0.048 | 0.023 | 0.07 | .947 |  |
|  | Gender: Man | -0.100 | -0.218 | 0.019 | 0.060 | -1.66 | .099 |  |
|  | Gender: Other | -0.023 | -0.235 | 0.189 | 0.108 | -0.21 | .831 |  |
|  | Country: UK | -0.043 | -0.129 | 0.043 | 0.044 | -0.98 | .328 |  |
| Negative tone | Well-Being | 0.010 | -0.041 | 0.062 | 0.026 | 0.40 | .687 | .894 |
|  | Ill-Being | 0.058 | 0.005 | 0.111 | 0.027 | 2.14 | .034* | .118 |
|  | Age | -0.057 | -0.107 | -0.007 | 0.025 | -2.27 | .025* |  |
|  | Gender: Man | 0.065 | -0.063 | 0.193 | 0.065 | 1.01 | .315 |  |
|  | Gender: Other | 0.095 | -0.132 | 0.322 | 0.115 | 0.82 | .411 |  |
|  | Country: UK | -0.071 | -0.163 | 0.022 | 0.047 | -1.50 | .135 |  |
| Positive emotion | Well-Being | 0.041 | 0.006 | 0.076 | 0.018 | 2.28 | .024* | .095 |
|  | Ill-Being | 0.030 | -0.007 | 0.067 | 0.019 | 1.58 | .115 | .249 |
|  | Age | 0.008 | -0.027 | 0.042 | 0.017 | 0.44 | .658 |  |
|  | Gender: Man | -0.006 | -0.094 | 0.082 | 0.045 | -0.13 | .900 |  |
|  | Gender: Other | 0.065 | -0.092 | 0.222 | 0.080 | 0.81 | .416 |  |
|  | Country: UK | -0.025 | -0.089 | 0.039 | 0.032 | -0.76 | .448 |  |
| Negative emotion | Well-Being | 0.023 | -0.021 | 0.068 | 0.022 | 1.04 | .300 | .560 |
|  | Ill-Being | 0.046 | 0.000 | 0.092 | 0.023 | 1.98 | .049* | .137 |
|  | Age | -0.032 | -0.075 | 0.011 | 0.022 | -1.49 | .138 |  |
|  | Gender: Man | 0.064 | -0.046 | 0.174 | 0.056 | 1.15 | .253 |  |
|  | Gender: Other | 0.082 | -0.114 | 0.277 | 0.099 | 0.83 | .410 |  |
|  | Country: UK | -0.133 | -0.213 | -0.053 | 0.040 | -3.30 | .001** |  |
| Anxiety | Well-Being | 0.028 | -0.004 | 0.060 | 0.016 | 1.71 | .090 | .209 |
|  | Ill-Being | 0.041 | 0.007 | 0.074 | 0.017 | 2.40 | .017* | .082 |
|  | Age | -0.029 | -0.060 | 0.002 | 0.016 | -1.84 | .068 |  |
|  | Gender: Man | 0.058 | -0.022 | 0.139 | 0.041 | 1.44 | .153 |  |
|  | Gender: Other | 0.017 | -0.126 | 0.159 | 0.072 | 0.23 | .818 |  |
|  | Country: UK | -0.072 | -0.130 | -0.014 | 0.029 | -2.45 | .016* |  |
| Anger | Well-Being | -0.008 | -0.030 | 0.015 | 0.011 | -0.68 | .500 | .737 |
|  | Ill-Being | 0.001 | -0.022 | 0.024 | 0.012 | 0.10 | .919 | .953 |
|  | Age | -0.008 | -0.029 | 0.013 | 0.011 | -0.77 | .442 |  |
|  | Gender: Man | 0.003 | -0.051 | 0.058 | 0.027 | 0.13 | .900 |  |
|  | Gender: Other | 0.073 | -0.027 | 0.172 | 0.050 | 1.45 | .150 |  |
|  | Country: UK | -0.065 | -0.104 | -0.025 | 0.020 | -3.23 | .002** |  |
| Sadness | Well-Being | 0.002 | -0.017 | 0.021 | 0.010 | 0.20 | .842 | .932 |
|  | Ill-Being | 0.009 | -0.011 | 0.029 | 0.010 | 0.86 | .391 | .608 |
|  | Age | -0.010 | -0.028 | 0.009 | 0.009 | -1.02 | .311 |  |
|  | Gender: Man | -0.019 | -0.067 | 0.029 | 0.024 | -0.79 | .429 |  |
|  | Gender: Other | 0.017 | -0.070 | 0.105 | 0.044 | 0.39 | .697 |  |
|  | Country: UK | -0.007 | -0.042 | 0.028 | 0.018 | -0.38 | .705 |  |

*Note.* **p* < .05, ** *p* < .01, ****p* < .001. LIWC = Linguistic Inquiry and Word Count. A sensitivity analysis was performed in which reports containing less than 25 words were excluded. In total, 125 dream reports containing <25 words were excluded and 1656 retained. Coefficients marked in bold remained significant after correcting for multiple comparisons with the Benjamini-Hochberg False Discovery Rate (Benjamini & Hochberg, 1995). Gender and country were entered using treatment (dummy) coding, with woman as the reference category for gender and Australia as the reference category for country. *N* = number of participants, *n* = number of reports.

**Supplementary Table 8**

*Results of Linear Mixed Effects Models Predicting Emotional Language Use (LIWC Categories) in Mind-Wandering Reports (N* = 152, *n* = 1496) *from Different Measures of Waking Well-Being and Ill-Being*

| LIWC  Category | Predictor | *B* | 95% CI LL | 95% CI UL | *SE* | *t* | *p* | Adjusted  *p* |
| --- | --- | --- | --- | --- | --- | --- | --- | --- |
| **Positive Tone** | SWLS | 0.049 | -0.011 | 0.109 | 0.030 | 1.62 | .107 | .112 |
|  | Age | 0.034 | -0.030 | 0.098 | 0.032 | 1.04 | .298 |  |
|  | Gender: Man | -0.056 | -0.225 | 0.113 | 0.085 | -0.66 | .513 |  |
|  | Gender: Other | -0.172 | -0.480 | 0.136 | 0.156 | -1.10 | .272 |  |
|  | Country: UK | 0.029 | -0.095 | 0.152 | 0.062 | 0.46 | .646 |  |
|  | BBQ | 0.051 | -0.010 | 0.111 | 0.031 | 1.66 | .099 | .110 |
|  | Age | 0.029 | -0.035 | 0.093 | 0.032 | 0.90 | .371 |  |
|  | Gender: Man | -0.062 | -0.231 | 0.106 | 0.085 | -0.73 | .468 |  |
|  | Gender: Other | -0.162 | -0.471 | 0.148 | 0.157 | -1.03 | .304 |  |
|  | Country: UK | 0.042 | -0.083 | 0.166 | 0.063 | 0.66 | .510 |  |
|  | PANAS-PA | 0.057 | -0.006 | 0.120 | 0.032 | 1.80 | .075 | .088 |
|  | Age | 0.018 | -0.048 | 0.083 | 0.033 | 0.54 | .592 |  |
|  | Gender: Man | -0.032 | -0.204 | 0.140 | 0.087 | -0.37 | .712 |  |
|  | Gender: Other | -0.179 | -0.486 | 0.127 | 0.155 | -1.16 | .250 |  |
|  | Country: UK | 0.010 | -0.115 | 0.135 | 0.063 | 0.16 | .875 |  |
|  | **PWB** | **0.108** | **0.050** | **0.165** | **0.029** | **3.70** | **< .001***** | **< .001***** |
|  | Age | 0.026 | -0.035 | 0.088 | 0.031 | 0.84 | .401 |  |
|  | Gender: Man | -0.014 | -0.179 | 0.150 | 0.083 | -0.17 | .862 |  |
|  | Gender: Other | -0.103 | -0.403 | 0.196 | 0.152 | -0.68 | .498 |  |
|  | Country: UK | 0.025 | -0.094 | 0.144 | 0.060 | 0.42 | .677 |  |
|  | **PoM-5** | **0.079** | **0.016** | **0.141** | **0.032** | **2.49** | **.014*** | **.020*** |
|  | Age | 0.012 | -0.053 | 0.077 | 0.033 | 0.36 | .717 |  |
|  | Gender: Man | -0.044 | -0.211 | 0.124 | 0.085 | -0.52 | .606 |  |
|  | Gender: Other | -0.158 | -0.463 | 0.146 | 0.154 | -1.03 | .306 |  |
|  | Country: UK | 0.037 | -0.085 | 0.159 | 0.062 | 0.60 | .550 |  |
| **Negative Tone** | **PANAS-NA** | **0.157** | **0.091** | **0.222** | **0.033** | **4.72** | **< .001***** | **< .001***** |
|  | Age | 0.069 | -0.001 | 0.139 | 0.035 | 1.95 | .054 |  |
|  | Gender: Man | 0.002 | -0.173 | 0.177 | 0.088 | 0.02 | .983 |  |
|  | Gender: Other | 0.009 | -0.309 | 0.326 | 0.161 | 0.05 | .957 |  |
|  | Country: UK | 0.055 | -0.074 | 0.183 | 0.065 | 0.84 | .401 |  |
|  | **GAD-7** | **0.157** | **0.094** | **0.220** | **0.032** | **4.91** | **< .001***** | **< .001***** |
|  | Age | 0.061 | -0.007 | 0.129 | 0.034 | 1.77 | .078 |  |
|  | Gender: Man | -0.007 | -0.181 | 0.166 | 0.088 | -0.08 | .933 |  |
|  | Gender: Other | 0.021 | -0.294 | 0.336 | 0.159 | 0.13 | .893 |  |
|  | Country: UK | 0.069 | -0.059 | 0.197 | 0.065 | 1.07 | .286 |  |
|  | **PHQ-9** | **0.140** | **0.076** | **0.205** | **0.033** | **4.30** | **< .001***** | **< .001***** |
|  | Age | 0.036 | -0.032 | 0.104 | 0.034 | 1.06 | .292 |  |
|  | Gender: Man | -0.037 | -0.215 | 0.141 | 0.090 | -0.41 | .681 |  |
|  | Gender: Other | -0.065 | -0.389 | 0.260 | 0.164 | -0.39 | .694 |  |
|  | Country: UK | 0.103 | -0.031 | 0.237 | 0.068 | 1.52 | .131 |  |
| **Negative Emotion** | **PANAS-NA** | **0.151** | **0.084** | **0.218** | **0.034** | **4.46** | **< .001***** | **< .001***** |
|  | Age | 0.062 | -0.009 | 0.134 | 0.036 | 1.72 | .088 |  |
|  | Gender: Man | -0.088 | -0.267 | 0.090 | 0.090 | -0.98 | .331 |  |
|  | Gender: Other | 0.014 | -0.311 | 0.339 | 0.164 | 0.09 | .932 |  |
|  | Country: UK | 0.057 | -0.074 | 0.189 | 0.066 | 0.86 | .389 |  |
|  | **GAD-7** | **0.158** | **0.093** | **0.222** | **0.033** | **4.84** | **< .001***** | **< .001***** |
|  | Age | 0.057 | -0.012 | 0.127 | 0.035 | 1.63 | .105 |  |
|  | Gender: Man | -0.096 | -0.273 | 0.080 | 0.089 | -1.08 | .283 |  |
|  | Gender: Other | 0.029 | -0.293 | 0.350 | 0.162 | 0.18 | .861 |  |
|  | Country: UK | 0.074 | -0.057 | 0.204 | 0.066 | 1.12 | .267 |  |
|  | **PHQ-9** | **0.131** | **0.065** | **0.197** | **0.033** | **3.95** | **< .001***** | **< .001***** |
|  | Age | 0.031 | -0.038 | 0.100 | 0.035 | 0.89 | .377 |  |
|  | Gender: Man | -0.124 | -0.306 | 0.057 | 0.092 | -1.35 | .178 |  |
|  | Gender: Other | -0.050 | -0.382 | 0.282 | 0.168 | -0.30 | .765 |  |
|  | Country: UK | 0.102 | -0.035 | 0.239 | 0.069 | 1.47 | .144 |  |
| **Anxiety** | **PANAS-NA** | **0.074** | **0.026** | **0.121** | **0.024** | **3.07** | **.003**** | **.005**** |
|  | Age | 0.004 | -0.046 | 0.055 | 0.026 | 0.17 | .864 |  |
|  | Gender: Man | -0.037 | -0.164 | 0.089 | 0.064 | -0.58 | .561 |  |
|  | Gender: Other | -0.028 | -0.259 | 0.202 | 0.116 | -0.24 | .807 |  |
|  | Country: UK | 0.112 | 0.020 | 0.205 | 0.047 | 2.39 | .018* |  |
|  | **GAD-7** | **0.088** | **0.043** | **0.133** | **0.023** | **3.84** | **< .001***** | **< .001***** |
|  | Age | 0.005 | -0.044 | 0.054 | 0.025 | 0.22 | .828 |  |
|  | Gender: Man | -0.041 | -0.166 | 0.083 | 0.063 | -0.66 | .513 |  |
|  | Gender: Other | -0.022 | -0.248 | 0.205 | 0.114 | -0.19 | .850 |  |
|  | Country: UK | 0.123 | 0.031 | 0.215 | 0.046 | 2.65 | .009** |  |
|  | **PHQ-9** | **0.055** | **0.008** | **0.102** | **0.024** | **2.34** | **.021*** | **.028*** |
|  | Age | -0.012 | -0.061 | 0.037 | 0.025 | -0.49 | .627 |  |
|  | Gender: Man | -0.053 | -0.182 | 0.076 | 0.065 | -0.81 | .418 |  |
|  | Gender: Other | -0.054 | -0.289 | 0.181 | 0.119 | -0.45 | .651 |  |
|  | Country: UK | 0.129 | 0.032 | 0.227 | 0.049 | 2.63 | .009** |  |
| **Anger** | **PANAS-NA** | **0.051** | **0.018** | **0.084** | **0.016** | **3.10** | **.002**** | **.005**** |
|  | Age | 0.010 | -0.025 | 0.044 | 0.017 | 0.56 | .573 |  |
|  | Gender: Man | -0.077 | -0.164 | 0.009 | 0.044 | -1.78 | .078 |  |
|  | Gender: Other | 0.008 | -0.149 | 0.165 | 0.079 | 0.10 | .923 |  |
|  | Country: UK | 0.062 | -0.001 | 0.126 | 0.032 | 1.94 | .054 |  |
|  | **GAD-7** | **0.047** | **0.015** | **0.079** | **0.016** | **2.94** | **.004**** | **.007**** |
|  | Age | 0.006 | -0.028 | 0.040 | 0.017 | 0.34 | .731 |  |
|  | Gender: Man | -0.081 | -0.167 | 0.006 | 0.044 | -1.85 | .066 |  |
|  | Gender: Other | 0.011 | -0.146 | 0.168 | 0.079 | 0.13 | .894 |  |
|  | Country: UK | 0.066 | 0.002 | 0.130 | 0.032 | 2.04 | .043* |  |
|  | PHQ-9 | 0.025 | -0.007 | 0.058 | 0.016 | 1.54 | .126 | .126 |
|  | Age | -0.004 | -0.038 | 0.029 | 0.017 | -0.25 | .800 |  |
|  | Gender: Man | -0.084 | -0.173 | 0.005 | 0.045 | -1.87 | .064 |  |
|  | Gender: Other | -0.005 | -0.168 | 0.157 | 0.082 | -0.07 | .948 |  |
|  | Country: UK | 0.067 | 0.000 | 0.135 | 0.034 | 1.98 | .049* |  |
| **Sadness** | **PANAS-NA** | **0.040** | **0.012** | **0.068** | **0.014** | **2.85** | **.005**** | **.009**** |
|  | Age | 0.030 | 0.000 | 0.059 | 0.015 | 1.99 | .049* |  |
|  | Gender: Man | -0.029 | -0.103 | 0.046 | 0.037 | -0.76 | .448 |  |
|  | Gender: Other | 0.001 | -0.133 | 0.136 | 0.068 | 0.02 | .986 |  |
|  | Country: UK | 0.008 | -0.047 | 0.062 | 0.028 | 0.28 | .780 |  |
|  | **GAD-7** | **0.030** | **0.002** | **0.057** | **0.014** | **2.13** | **.035*** | **.044*** |
|  | Age | 0.024 | -0.005 | 0.054 | 0.015 | 1.64 | .105 |  |
|  | Gender: Man | -0.031 | -0.106 | 0.044 | 0.038 | -0.82 | .411 |  |
|  | Gender: Other | 0.003 | -0.134 | 0.139 | 0.069 | 0.04 | .970 |  |
|  | Country: UK | 0.008 | -0.047 | 0.064 | 0.028 | 0.29 | .770 |  |
|  | **PHQ-9** | **0.037** | **0.009** | **0.064** | **0.014** | **2.65** | **.009**** | **.014*** |
|  | Age | 0.021 | -0.008 | 0.049 | 0.014 | 1.45 | .151 |  |
|  | Gender: Man | -0.039 | -0.114 | 0.036 | 0.038 | -1.02 | .308 |  |
|  | Gender: Other | -0.021 | -0.157 | 0.115 | 0.069 | -0.31 | .759 |  |
|  | Country: UK | 0.020 | -0.036 | 0.077 | 0.029 | 0.71 | .479 |  |

*Note.* **p* < .05, ** *p* < .01, ****p* < .001. Coefficients marked in bold remained significant after correcting for multiple comparisons with the Benjamini-Hochberg False Discovery Rate (Benjamini & Hochberg, 1995). Gender and country were entered using treatment (dummy) coding, with woman as the reference category for gender and Australia as the reference category for country. *N* = number of participants, *n* = number of reports. IB = ill-being, WB = well-being. LIWC = Linguistic Inquiry and Word Count. *N* = number of participants, *n* = number of reports. SWLS = Satisfaction with Life Scale. BBQ = Brunnsviken Brief Quality of Life Scale (BBQ). PANAS = Positive Affect Negative Affect Schedule. PA = positive affect. NA = negative affect. PWB = Ryff's Scales of Psychological Well-Being. PoM-5 = Peace of Mind Scale. PHQ-9 = Depression scale from Patient Health Questionnaire. GAD-7 = Generalized Anxiety Disorder Scale.

**Supplementary Table 9**

*Results of Linear Mixed Effects Models Predicting Emotional Language Use (LIWC Categories) in Dream Reports (N* = 172, *n* = 1781) *from Different Measures of Waking Well-Being and Ill-Being*

| LIWC Category | Predictor | *B* | 95% CI LL | 95% CI UL | *SE* | *t* | *p* | Adjusted  *p* |
| --- | --- | --- | --- | --- | --- | --- | --- | --- |
| **Anxiety** | **PANAS-NA** | **0.042** | **0.012** | **0.072** | **0.015** | **2.78** | **.006**** | **.018*** |
|  | Age | -0.017 | -0.049 | 0.014 | 0.016 | -1.08 | .282 |  |
|  | Gender: Man | 0.038 | -0.042 | 0.118 | 0.040 | 0.95 | .346 |  |
|  | Gender: Other | -0.011 | -0.153 | 0.131 | 0.072 | -0.16 | .877 |  |
|  | Country: UK | -0.092 | -0.150 | -0.034 | 0.029 | -3.15 | .002** |  |
|  | GAD-7 | 0.031 | 0.001 | 0.060 | 0.015 | 2.07 | .040* | .060 |
|  | Age | -0.023 | -0.055 | 0.009 | 0.016 | -1.44 | .153 |  |
|  | Gender: Man | 0.033 | -0.047 | 0.114 | 0.041 | 0.82 | .415 |  |
|  | Gender: Other | -0.013 | -0.157 | 0.132 | 0.073 | -0.17 | .864 |  |
|  | Country: UK | -0.092 | -0.151 | -0.033 | 0.030 | -3.08 | .002** |  |
|  | PHQ-9 | 0.024 | -0.006 | 0.053 | 0.015 | 1.58 | .117 | .117 |
|  | Age | -0.029 | -0.060 | 0.001 | 0.016 | -1.90 | .060 |  |
|  | Gender: Man | 0.027 | -0.055 | 0.109 | 0.041 | 0.66 | .510 |  |
|  | Gender: Other | -0.028 | -0.175 | 0.119 | 0.074 | -0.38 | .704 |  |
|  | Country: UK | -0.084 | -0.145 | -0.023 | 0.031 | -2.74 | .007** |  |

*Note.* **p* < .05, ** *p* < .01, ****p* < .001. Coefficients marked in bold remained significant after correcting for multiple comparisons with the Benjamini-Hochberg False Discovery Rate (Benjamini & Hochberg, 1995). Gender and country were entered using treatment (dummy) coding, with woman as the reference category for gender and Australia as the reference category for country. *N* = number of participants, *n* = number of reports. IB = ill-being, WB = well-being. LIWC = Linguistic Inquiry and Word Count. *N* = number of participants, *n* = number of reports. SWLS = Satisfaction with Life Scale. BBQ = Brunnsviken Brief Quality of Life Scale (BBQ). PANAS = Positive Affect Negative Affect Schedule. PA = positive affect. NA = negative affect. PWB = Ryff's Scales of Psychological Well-Being. PoM-5 = Peace of Mind Scale. PHQ-9 = Depression scale from Patient Health Questionnaire. GAD-7 = Generalized Anxiety Disorder Scale.

**Supplementary Table 10**

*Linear Mixed-Effects Models Examining Within- and Between-Person Associations Between Self-Rated Affect and Emotional Language Use in Mind-Wandering Reports (N = 152, n = 1496) and in Dream Reports (N = 172, n = 1781)*

| Dataset | LIWC Category | Self-Rated Affect | Level | *B* | 95% CI LL | 95% CI UL | *SE* | *t* | *p* | Adjusted  *p* |
| --- | --- | --- | --- | --- | --- | --- | --- | --- | --- | --- |
| Dreams | Negative tone | Negative affect | Within-person | 0.233 | 0.203 | 0.264 | 0.016 | 14.83 | < .001*** | < .001*** |
|  | Negative tone | Negative affect | Between-person | 0.258 | 0.199 | 0.318 | 0.030 | 8.54 | < .001*** | < .001*** |
|  | Negative emotion | Negative affect | Within-person | 0.176 | 0.150 | 0.202 | 0.013 | 13.24 | < .001*** | < .001*** |
|  | Negative emotion | Negative affect | Between-person | 0.208 | 0.155 | 0.260 | 0.026 | 7.86 | < .001*** | < .001*** |
|  | Positive tone | Positive affect | Within-person | 0.183 | 0.149 | 0.217 | 0.017 | 10.53 | < .001*** | < .001*** |
|  | Positive tone | Positive affect | Between-person | 0.162 | 0.088 | 0.237 | 0.038 | 4.30 | < .001*** | < .001*** |
|  | Positive emotion | Positive affect | Within-person | 0.131 | 0.108 | 0.155 | 0.012 | 11.17 | < .001*** | < .001*** |
|  | Positive emotion | Positive affect | Between-person | 0.115 | 0.068 | 0.162 | 0.024 | 4.79 | < .001*** | < .001*** |
| Mind-wandering | Negative tone | Negative affect | Within-person | 0.277 | 0.242 | 0.312 | 0.018 | 15.59 | < .001*** | < .001*** |
|  | Negative tone | Negative affect | Between-person | 0.425 | 0.342 | 0.508 | 0.042 | 10.10 | < .001*** | < .001*** |
|  | Negative emotion | Negative affect | Within-person | 0.263 | 0.231 | 0.295 | 0.016 | 16.19 | < .001*** | < .001*** |
|  | Negative emotion | Negative affect | Between-person | 0.410 | 0.323 | 0.497 | 0.044 | 9.28 | < .001*** | < .001*** |
|  | Positive tone | Positive affect | Within-person | 0.194 | 0.158 | 0.230 | 0.018 | 10.55 | < .001*** | < .001*** |
|  | Positive tone | Positive affect | Between-person | 0.227 | 0.144 | 0.311 | 0.042 | 5.36 | < .001*** | < .001*** |
|  | Positive emotion | Positive affect | Within-person | 0.165 | 0.131 | 0.198 | 0.017 | 9.63 | < .001*** | < .001*** |
|  | Positive emotion | Positive affect | Between-person | 0.178 | 0.097 | 0.259 | 0.041 | 4.34 | < .001*** | < .001*** |

*Note.* ****p* < .001. *N* = number of participants, *n* = number of reports. LIWC = Linguistic Inquiry and Word Count. Separate linear mixed-effects models were fitted for each LIWC outcome in each report type (eight models in total), with a random intercept for participant and no covariates. Each affect rating was decomposed into a within-person component (report score minus that participant's mean) and a between-person component (that participant's mean), and used to predict the valence-matched LIWC categories. LIWC outcome variables were square-root transformed. Predictors are unstandardized, so B is the change per 1-point change in the affect rating. Adjusted p-values are Benjamini-Hochberg False Discovery Rate corrections (Benjamini & Hochberg, 1995).

**Supplementary Figure 1**

*Principal Component Analysis Biplot*

*Note*. The biplot displays the first two principal components (PC1 and PC2) derived from the dataset, explaining 51.4% and 18.7% of the total variance, respectively. Points represent individual participants, with transparency indicating overlap. Arrows indicate the loadings of the original variables, showing their contributions to the principal components, with the direction and length representing the strength and influence of each variable on PC1 and PC2, and are colored according to whether the scale contributes to the well-being or the ill-being composite. Variable labels are positioned near their respective arrows. SWLS = Satisfaction with Life Scale. BBQ = Brunnsviken Brief Quality of Life Scale (BBQ). PANAS = Positive Affect Negative Affect Schedule. PA = positive affect. NA = negative affect. PWB = Ryff's Scales of Psychological Well-Being. PoM-5 = Peace of Mind Scale. PHQ-9 = Depression scale from Patient Health Questionnaire. GAD-7 = Generalized Anxiety Disorder Scale.
